# Brominated Methanesulfonates: Characterization of K[Br_3_CSO_3_] ⋅ H_2_O, K_2_[Br_2_C(SO_3_)_2_] ⋅ H_2_O and K_3_[BrC(SO_3_)_3_] ⋅ H_2_O

**DOI:** 10.1002/open.202500455

**Published:** 2025-11-05

**Authors:** Katrin Eppers, Celina Sander, D. van Gerven, Mathias S. Wickleder

**Affiliations:** ^1^ Universität zu Köln Institut für Anorganische Chemie Greinstraße 6 50939 Köln Germany

**Keywords:** bromination, crystal structure, methanesulfonic acid, polysulfonates, theory

## Abstract

Bromination of phenyl methanesulfonate, C_6_H_5_OSO_2_CH_3_, with KOBr followed by hydrolytic cleavage of the phenyl ester leads to the tribromomethanesulfonate (“tribrate”) K[Br_3_CSO_3_] ⋅ H_2_O, which crystallizes in a hitherto unknown triclinic modification (*P* $\overset{―}{1}$, *a* = 662.87(4) pm, *b* = 1090.98(7) pm, *c* = 1273.98(8) pm, *α *= 106.079(2)°, *β *= 93.438(2)°, *γ* = 90.121(2)°). In contrast to the synthesis of the tribrate, in which an aromatic ring must be present at the SO_3_ group for a successful bromination, the synthesis of K_2_[Br_2_C(SO_3_)_2_] ⋅ H_2_O (monoclinic, *P*2_1_/*c*, *Z* = 4, *a* = 717.17(3) pm, *b* = 710.78(3) pm, *c* = 2092.50(9) pm, *β* = 94.732(2)°) and K_3_[BrC(SO_3_)_3_] ⋅ H_2_O (tetragonal, *P*4_3_, *Z* = 4, *a* = 713.6(1) pm, *c* = 2324.50(7) pm) using KOBr do not need the phenylesters as starting materials. Comparing the C—S bond lengths of the different anions with each other, a trend emerges in which the C—S bond length increases with increasing number of SO_3_ groups (decreasing number of Br atoms). Furthermore, an increase in thermal stability by ≈50 °C per additional SO_3_ group can be observed. The compounds are characterized by X‐ray diffraction, vibrational spectroscopy, and thermal analyses.

## Graphical abstract

The sulfonates of methane can be easily brominated using hypobromite in aqueous solution. The structures of resulting anions [Br_3_CSO_3_]^−^, [Br_2_C(SO_3_)_2_]^2−^, and [BrC(SO_3_)_3_]^3−^ can be analysed for the respective potassium salts.

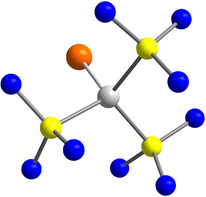

## 1 Introduction

The simplest organic sulfonic acids are based on methane, CH_4_. Formally, the acids are obtained by insertion of SO_3_ into the C—H bonds of CH_4_. Accordingly, the molecules CH_3_SO_3_H (methanesulfonic acid), CH_2_(SO_3_H)_2_ (methanedisulfonic acid), CH(SO_3_H)_3_ (methanetrisulfonic acid), and C(SO_3_H)_4_ (methanetetrasulfonic acid) are possible derivatives. Methanesulfonic acid is well known and widely used because it is a strong acid but less oxidizing than sulfuric acid, and, furthermore, it is a biodegradable compound.^[^ ^1^, ^2^ ^–^ ^3^ ^]^ Especially in the treatment of metal waste and as an electrolyte, the acid has attracted attention.^[^ ^4^ ^,^ ^5^ ^]^ The di‐ and trisulfonic acids are also known, while methanetetrasulfonic acid is still elusive. For CH_3_SO_3_H and CH_2_(SO_3_H)_2_, the crystal structures of the neat acids have been elucidated.^[^ ^6^ ^,^ ^7^ ^]^ CH(SO_3_H)_3_ is structurally only known as its trihydrate, an oxonium salt according to (H_3_O)_3_[CH(SO_3_)_3_],^[^ ^8^ ^]^ which has been shown to be a versatile catalyst.^[^ ^9^ ^]^ For all three acids, a number of different salts have been reported, and several crystal structures are known.^[^ ^8^ ^,^ ^10^, ^11^, ^12^, ^13^, ^14^, ^15^, ^16^, ^17^, ^18^, ^19^, ^20^, ^21^, ^22^, ^23^, ^24^, ^25^, ^26^, ^27^, ^28^, ^29^, ^30^, ^31^, ^32^, ^33^, ^34^, ^35^, ^36^ ^–^ ^37^ ^]^ As expected, the majority of described compounds are the salts of methanesulfonic acid, CH_3_SO_3_H. A highly important derivative of CH_3_SO_3_H is trifluoromethanesulfonic acid, CF_3_SO_3_H (‘triflic acid’). It is a very strong Brönsted acid and its salts, the so‐called triflates, are frequently used, for example, as electrolytes and Lewis acid catalysts. CF_3_SO_3_H is prepared by an electrochemical fluorination process.^[^ ^38^ ^]^ The same method is used to prepare also the fluorinated derivative of CH_2_(SO_3_H)_2_, namely CF_2_(SO_3_H)_2_,^[^ ^39^ ^]^ for which the structures of some salts have been reported.^[^ ^40^ ^]^ A fluoro derivative of methanetrisulfonic acid is not known. First reports for sulfonic acid derivatives of the higher halogens Cl, Br, and I originate already from the end of the 19th century. Further investigations were undertaken in 1929/30, especially by Backer et al. In the 1990s and at the beginning of the 21st century, the class of methane(poly)sulfonic acids and their halogen derivatives attracted renewed interest. However, structural proofs for these compounds are still scarce. Only for the trichloromethanesulfonate anion (‘trichlate’), [Cl_3_CSO_3_]^−^, two independent investigations were reported in 2006.^[^ ^41^ ^,^ ^42^ ^]^ Recently, we started to investigate in more detail the polysulfonic acids of methane. The work was a continuation of former investigations on polysulfonic acids with larger and more complex organic scaffolds.^[^ ^43^ ^]^ In the course of our work, we were not only able to enlarge the group of trichlates,^[^ ^44^, ^45^ ^–^ ^46^ ^]^ but we also prepared for the first time the tribromomethanesulfonate anion, [Br_3_CSO_3_]^−^, which we named the ‘tribrate’ anion.^[^ ^47^ ^]^ The synthesis was done by the reaction of the phenyl ester of methanesulfonic acid and hypobromite, KOBr, and subsequent splitting of the ester, yielding monoclinic K[Br_3_CSO_3_] ⋅ H_2_O. It has now been discovered that this bromination reaction is also an elegant method for producing the anion [Br_2_C(SO_3_)_2_]^2−^ and [BrC(SO_3_)_3_]^3−^. This avoids high‐pressure reactions using elemental chlorine or bromine, which have previously been reported to be necessary.^[^ ^48^ ^]^ Here, we present for the first time the structures of both anions as found in their potassium salts, along with a new modification of K[Br_3_CSO_3_] ⋅ H_2_O.

## 2 Results and Discussion

### 2.1 Syntheses

**Scheme 1:**
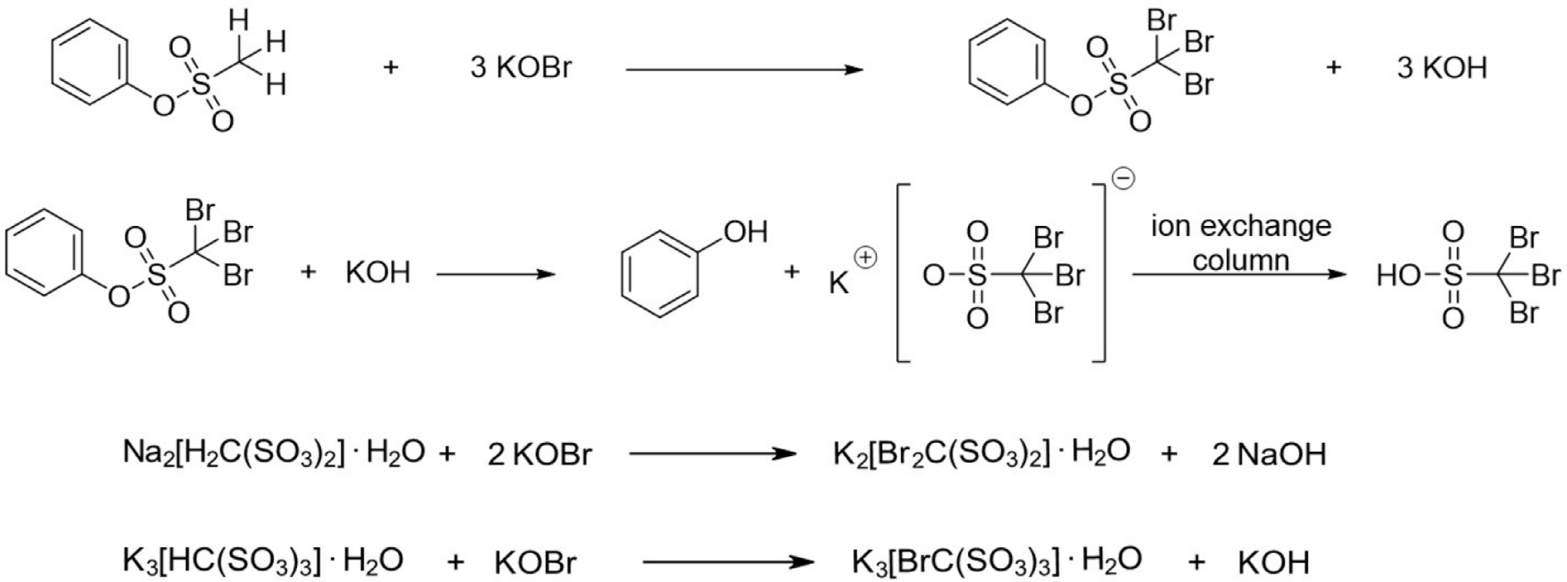
The synthesis of [Br_3_CSO_3_]^−^ is shown in the two upper lines. The two equations below describe the syntheses of [Br_2_C(SO_3_)_2_]^2−^ and [BrC(SO_3_)_3_]^3−^, respectively.

The reaction for the bromination of a CH_3_ group by hypobromite was described by Ochal et al. in 2012 and Stevens et al. in 2014. The first group could show that the CH_3_ group of aryl methylsulfones, ArSO_2_CH_3_ (Ar = Aryl), can be brominated by sodium hypobromite, NaOBr, even in aqueous solution.^[^ ^49^ ^]^ Later, Stevens et al. proved that the related sulfonates, ArOSO_2_CH_3_, can be brominated under these conditions.^[^ ^50^ ^]^ For the synthesis of K[Br_3_CSO_3_] ⋅ H_2_O, we adopted this approach.^[^ ^47^ ^]^ Thus, we have reacted the phenyl ester of methanesulfonic acid with KOBr at 65 °C. The brominated ester was obtained with 69% yield after extraction with Et_2_O (**Scheme** **1**). After the reaction, the brominated ester has been cleaved under basic conditions, and the acid could be obtained using an ion exchange column.

Previous investigations suggest that the bromination of the methanedisulfonate and ‐trisulfonate anion can be achieved by reactions with elemental bromine under elevated pressure.^[^ ^31^ ^,^ ^48^ ^]^ Here, we adopted the bromination reaction using hypobromite also for the [CH_2_(SO_3_)_2_]^2−^ and [CH(SO_3_)_3_]^3−^ anions. It turned out that for these compounds, the formation of esters as starting materials is not necessary. Accordingly, the reactions were carried out in aqueous solutions starting with methanedisulfonate and ‐trisulfonate salts. The brominated disulfonate could be obtained at 70 °C with a yield of 47% while the trisulfonate forms at 45 °C with a 78% yield. Obviously, the acidity of the involved hydrogen atoms plays an important role in the reaction mechanism. The details of the syntheses can be found in the supporting information.

### 2.2 Crystal Structures

**Figure 1:**
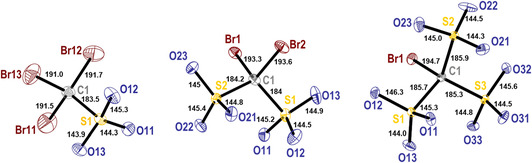
The anions [Br_3_CSO_3_]^−^ (left), [Br_2_C(SO_3_)_2_]^2−^ (middle), and [BrC(SO_3_)_3_]^3−^ (right) as found in the crystal structures of their potassium salts. The thermal ellipsoids are set to a 70% probability level. Distances are given in pm.

The new modification of K[Br_3_CSO_3_] ⋅ H_2_O crystallizes with triclinic symmetry and shows two crystallographically different [Br_3_CSO_3_]^−^ ions. Compared to the recently reported monoclinic K[Br_3_CSO_3_] ⋅ H_2_O modification, the triclinic polymorph has a slightly higher density (2.924 vs. 2.914 g cm^−3^). Accordingly, we name the new triclinic modification K[Br_3_CSO_3_] ⋅ H_2_O‐I. In the crystal structure, both anions have a staggered conformation with respect to the orientation of the [CBr_3_] and [SO_3_] groups. The anions show almost the same bond lengths and angles (see supporting information for full data), and one of the anions is shown in the left part of **Figure** **1**. Thus, the S—O distances are found for both ions between 144 and 145 pm, while the S—C bond shows values of 183.5(5) and 184.6(5) pm. The C—Br distances range from 189.7(5) to 193.4(5) pm. It is interesting that the longest C—Br distance correlates to the shortest K—Br distance in the structure (K2—Br23), showing that there is a small contribution of the [CBr_3_] moiety to the K^+^ coordination.

**Figure 2:**
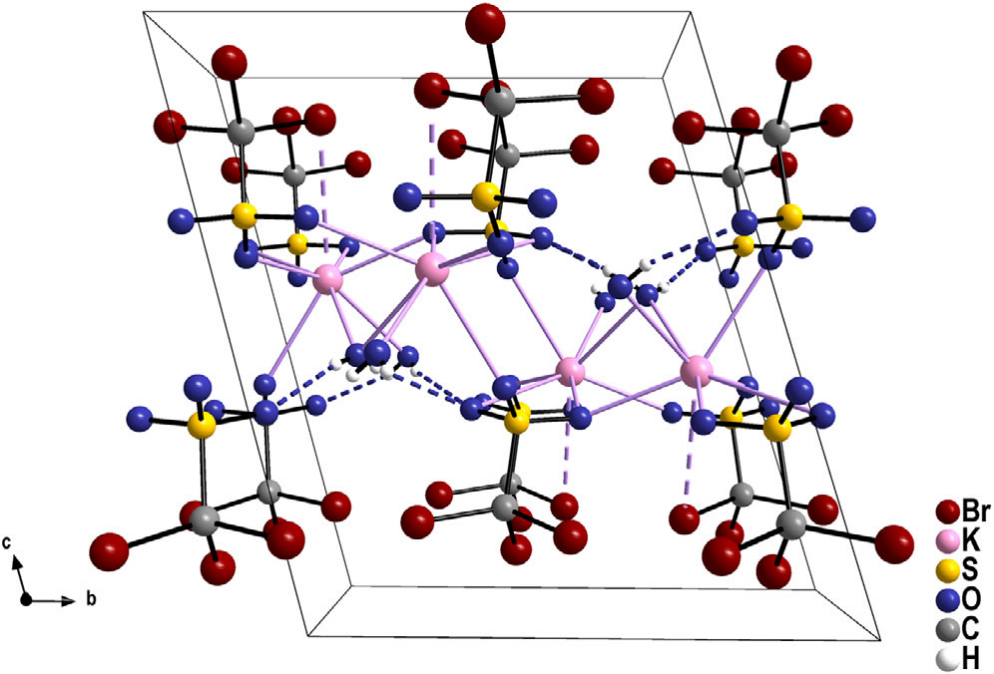
Perspective representation of the crystal structure of K[Br_3_CSO_3_] ⋅ H_2_O viewed along the [100] direction. The K^+^ ions are coordinated by seven oxygen atoms and one bromine atom with long distances at 371 and 377 pm, emphasized as hatched purple lines. Hydrogen bonds are shown as blue hatched lines.

Both K^+^ ions in the crystal structure of K[Br_3_CSO_3_] ⋅ H_2_O are coordinated by seven oxygen atoms with the distances K—O ranging from 270.7(3) to 317.2(4) pm. The oxygen atoms belong to the [SO_3_] groups of the anions and to the crystal water molecule in the structure. Furthermore, both K^+^ ions have a weak contact to bromine atoms of the anions with distances of 371.3(1) and 377.5(1) pm. The [SO_3_] moieties, the H_2_O molecules, and the K^+^ ions are arranged to layers which are oriented parallel to the *a–b* plane of the triclinic lattice (**Figure** **2**). The H_2_O molecules are bonded to the K^+^ ions and show additionally weak hydrogen bonds to the oxygen atoms of adjacent [SO_3_] groups (see supporting information). The oxygen‐rich layers alternate with layers formed by the [CBr_3_] groups of the anion. Such layer‐type structures with an alternation of hydrophilic and hydrophobic areas are quite typical for the structures of triflates. Interestingly, the structure of K[F_3_CSO_3_] is very different to the presented structure of K[Br_3_CSO_3_] ⋅ H_2_O.^[^ ^51^ ^]^

**Figure 3:**
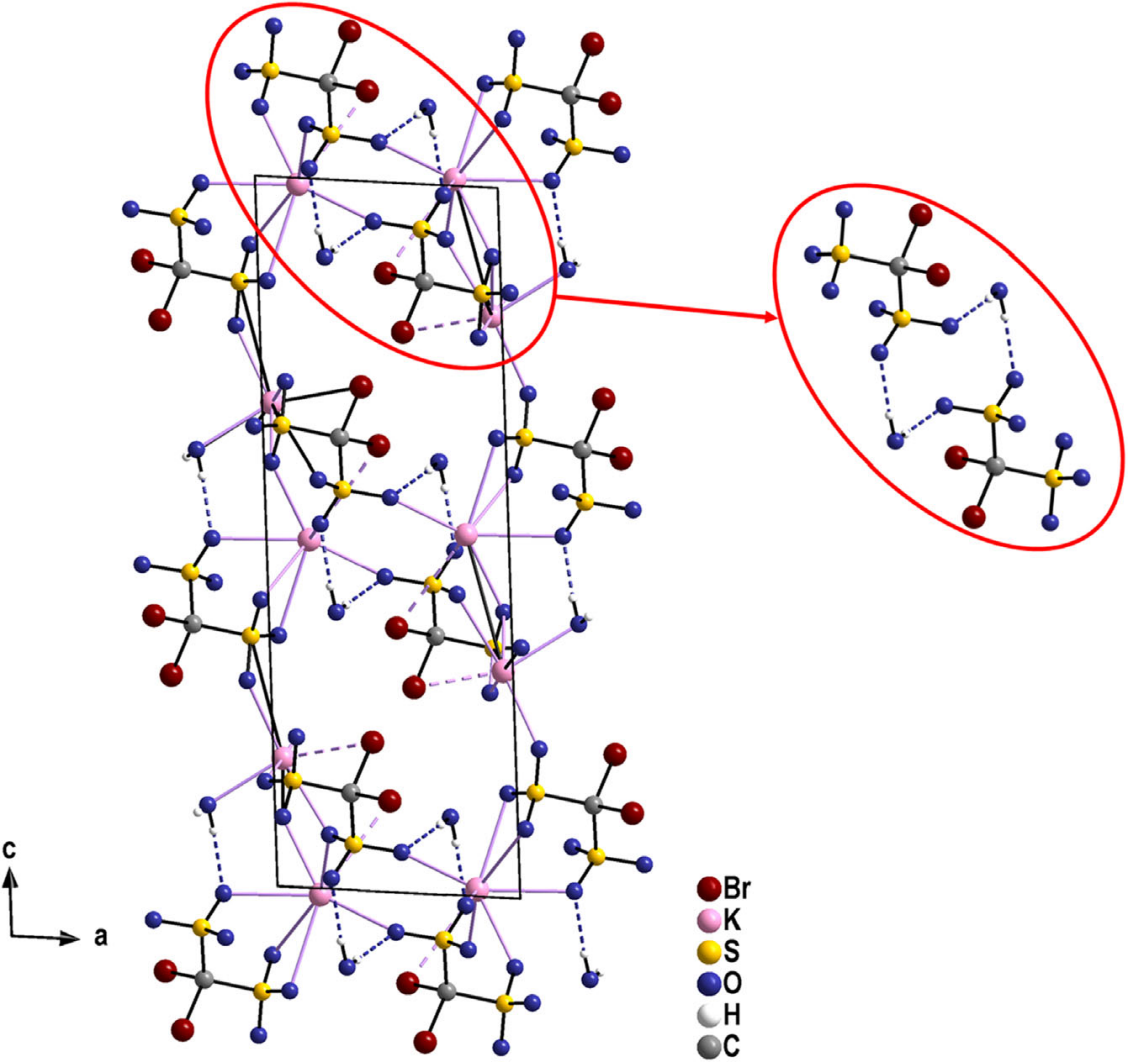
Projection of the crystal structure K_2_[Br_2_C(SO_3_)_2_] ⋅ H_2_O onto the (010) plane (left). The K^+^ ions are coordinated by seven oxygen atoms and one bromine atom with long distances at 343 and 358 pm, emphasized as hatched purple lines. Hydrogen bonds are shown as blue hatched lines. The hydrogen bonds of the H_2_O molecules occur between two adjacent anions (right).

The new [Br_2_C(SO_3_)_2_]^2−^ anion in the monoclinic structure of K_2_[Br_2_C(SO_3_)_2_] ⋅ H_2_O is situated on a general site (4*e*) leading to *C*_1_ symmetry for the anion. In an ideal case, the anion may adopt *C*_2v_ symmetry, and the deviation from this symmetry is caused essentially by the orientation of the [SO_3_] groups with respect to each other, as can be seen from the torsion angles O—S—S—O of about 12°. Within the anion, the S—O distances show similar values ranging from 144.0(2) to 145.4(2) pm. The S—C bond lengths are both 184.2(2) and 184.2(2) pm, and the C—Br bonds in the [CBr_2_] group show almost the same distances with values of 193.3(2) and 193.6(2) pm. Both bromine atoms are involved in the coordination of the K^+^ ions with K—Br distances of 343.10(5) and 358.92(6) pm. Furthermore, the K^+^ ions are coordinated by six oxygen atoms of the [SO_3_] groups and the crystal water molecules (**Figure** **3**, left). The crystal water molecules are involved in some weak, moderate hydrogen bonds to oxygen atoms of the [SO_3_] moieties. In this way, adjacent [Br_2_C(SO_3_)_2_]^2−^ anions are bridged into dimers (Figure 3, right).

**Figure 4:**
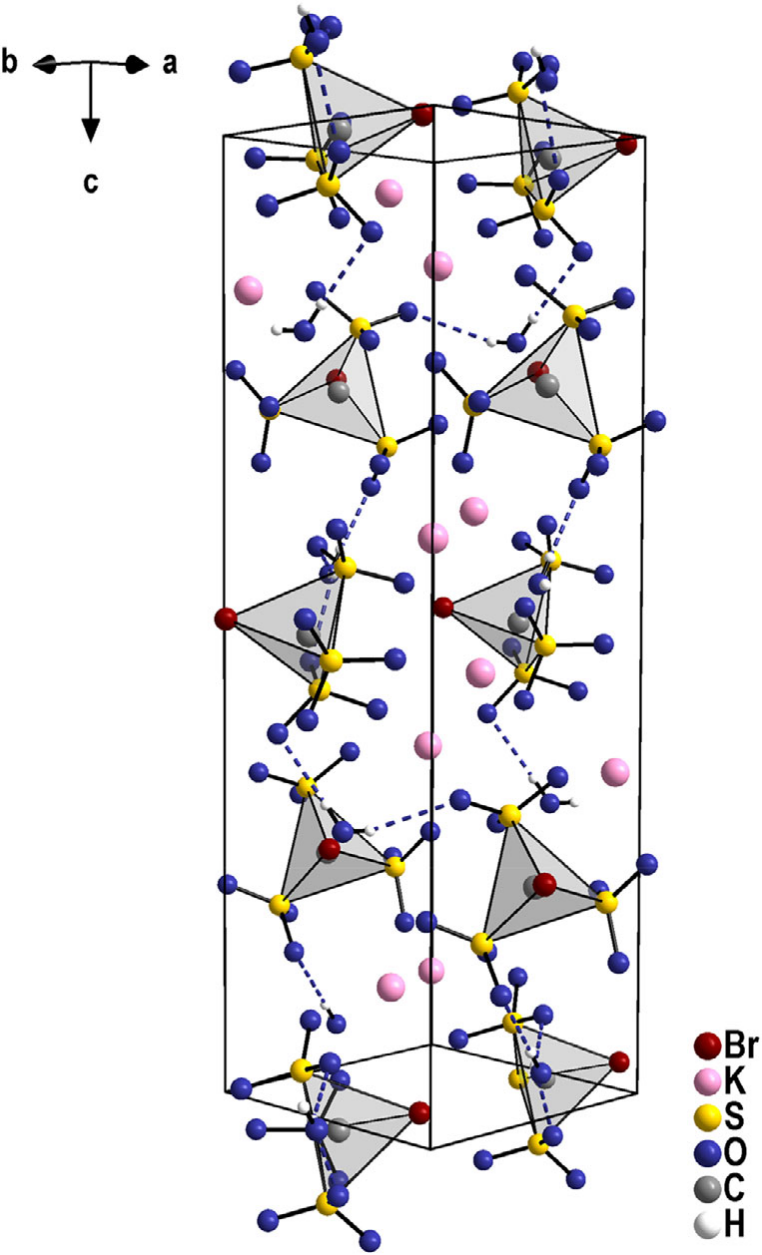
Perspective view of the K_3_[BrC(SO_3_)_3_] ⋅ H_2_O along the [110] direction. The [CS_3_Br] tetrahedra are shown as polyhedra and display nicely the 4_3_ screw axis in the crystal structure. Important hydrogen bonds are shown as blue hatched lines.

The structure of K_3_[BrC(SO_3_)_3_] ⋅ H_2_O is tetragonal with the non‐centrosymmetric and chiral space group *P*4_3_. The structure allows the detailed analyses of the [BrC(SO_3_)_3_]^3−^ anion for the first time. There is one distinct anion in the crystal structure, which is situated on a general site of space group *P*4_3_. Thus, the symmetry of the anion is *C*_1_ in the investigated compound, while the ideal symmetry should be *C*_3v_. In the anion, the C—S bonds are found in a narrow range around 185.5 pm. The S—O bond lengths occur between 144.3(3) and 145.6(3) pm, and the C—Br bond shows a value of 194.7(3) pm. The bromine atom takes part in the coordination of the K^+^ ion with distances between 352.0(8) and 369.9(8) pm. Interestingly, the three crystallographically different K^+^ ions participate quite differently in the bromine coordination. One of the cations has contact with three bromine atoms, the second one with one Br atom, while the third cation is only attached to oxygen atoms (Table S22, Supporting Information). For the cation, this coordination results in a coordination number between 8 and 10. The crystal water molecule participates in the coordination of the K^+^ ions and is furthermore involved in some weak hydrogen bonds to oxygen atoms of the [SO_3_] moieties of the anions. In the crystal structure, the anions are arranged along the *c*‐axis of the unit cell and illustrate nicely the 4_3_ axis of the space group (**Figure** **4**).

### 2.3 Comparison of the Anions

All of the new bromomethanesulfonates were obtained as potassium salts **Table** **1**. Thus, it can be assumed that the influence of the cation on the structural features of the anion is similar for all compounds, and a reliable comparison of the anions is possible. The S—O distances within the [SO_3_] groups of the anions do not depend on the number of bromine atoms. They are found uniformly at an average distance of about 145 pm (**Table** **2**). The distances S—C increase slightly but not significantly with the number of sulfonate groups. The largest differences are observed for the C—Br distances, which increase from an average value of 191.6 pm in the tribrate to 193.4 pm in the dibromomethanedisulfonate up to 194.7 pm in the trisulfonate. Theoretical studies for the anions at the density functional theory (DFT) level also predict only slow variations in the bond lengths of the anions with increasing sulfonation degree (see supporting information). It should be interesting to compare the findings for the bromo derivatives with the respective chloro‐, fluoro‐, and hydrogen derivatives. The respective potassium compounds are known for K[CH_3_SO_3_],^[^ ^52^ ^]^ K_2_[CH_2_(SO_3_)_2_],^[^ ^32^ ^]^ K_3_[CH(SO_3_)_3_](H_2_O),^[^ ^8^ ^]^ K[F_3_CSO_3_],^[^ ^49^ ^]^ K_2_[F_2_C(SO_3_)_2_],^[^ ^40^ ^]^ K[Cl_3_CSO_3_],^[^ ^53^ ^]^ and K_3_[ClC(SO_3_)_3_](H_2_O).^[^ ^53^ ^]^ It is obvious that the [SO_3_] moieties show essentially the same bonding parameters in all compounds. The differences in the C—S distances are most significant for the hydrogen compounds. With increasing sulfonation, the distances increase from 175.1 in the [CH_3_SO_3_]^−^ ion to 181.9 pm in [HC(SO_3_)_3_]^3−^. This can be attributed to the electron‐withdrawing of the [SO_3_] groups. The substitution of a hydrogen by a halogen atom leads to another lengthening of the S—C bond. Between [H_3_CSO_3_]^−^ and [F_3_CSO_3_]^−^ this is significant with an increase of the S—C bond length of about 8 pm. Interestingly, the distances for the trichlate (184.3 pm) and tribrate (184.0 pm) anion are essentially identical to the findings for the triflate anion. Thus, the halogenation of the methanesulfonate anion leads to a growth of the S—C bond length, but the lengthening is obviously not dependent on the electronegativity of the halogen atom. For the halogenated species, the number of [SO_3_] groups has no significant effect on the S—C bond. Thus, the changes of the S—C bond in the row [X_3_CSO_3_]^−^, [X_2_C(SO_3_)_2_]^2−^, [XC(SO_3_)_3_]^3−^ seem to be quite small. However, at least for the [BrC(SO_3_)_3_]^3−^ anion, the increase of the S—C bond is significant. Unfortunately, the [FC(SO_3_)_3_]^3−^ ion and the [Cl_2_C(SO_3_)_2_]^2−^ are not known up to now, so that a clear picture is still elusive.

**Table 1:** Crystallographic data of the investigated compounds.

Compounda)	K[Br_3_CSO_3_] ⋅ H_2_O	K_2_[Br_2_C(SO_3_)_2_] ⋅ H_2_O	K_3_[BrC(SO_3_)_3_] ⋅ H_2_O
[*T * *K*]	100	100	100
Crystal System	*triclinic*	*monoclinic*	*tetragonal*
Space group	*P* $\overset{―}{1}$	*P*2_1 _ *c*	*P*4_3_
*a *[pm]	662.87(4)	717.17(3)	713.6(1)
*b *[pm]	1090.98(7)	710.78(3)	–
*c *[pm]	1273.98(8)	2092.50(9)	2324.50(7)
*α *[°]	106.079(2)	–	–
*β *[°]	93.438(2)	94.732(2)	–
*γ *[°]	90.121(2)	–	–
V [Å^−3^]	883.5(1)	1063.02(8)	1183.79(5)
*Z*	4	8	4
Final *R* indexes [all data]	*R*_1 _= 0.0543, *wR*_2 _= 0.1186	*R*_*1*_ = 0.0276, *wR*_*2 *_= 0.0649	*R*_*1*_ = 0.0170, *wR*_*2*_ = 0.0385
*R*_int_	0.0563	0.0442	0.0584
Goof	1.095	1.186	1.153
Flack‐x	–	–	0.001(3)
CCDC number	2386367	2341531	2341533

a) full data can be found in the Supporting Information.

**Table 2:** Comparison of the bond lengths in methanesulfonates and their halogenated derivatives (average distances are given in brackets).

Bond	*X*	[X_3_CSO_3_]^−^	[X_2_C(SO_3_)_2_]^2−^	[XC(SO_3_)_3_]^3−^
S—O	–	144.1–146.2 (145.2)	144.8–147.0 (146.2)	144.4–148.4 (145.4)
S—C	H	175.0–175.2 (175.1)	177.0	179.5–185.1 (181.9)
C—X	–	85–100 (91)	104	100
				
S—O	–	144.0–150.9 (146.0)	142.7–144.6 (143.9)	–
S—C	F	181.2–185.5 (183.8)	184.0–184.2 (184.1)	–
C—X	–	129.9–149.2 (140.1)	133.7–135.8 (134.8)	–
				
S—O	–	144.5–145.3 (144.9)	–	138.6–154.8 (144.8)
S—C	Cl	183.9–184.6 (184.3)	–	183.3–183.4 (183.35)
C—X	–	175.7–177.0 (176.5)	–	179.8
				
S—O	–	143.9–145.3 (144.6)	144.5–145.4 (144.9)	144.3–146.3 (144.9)
S—C	Br	183.5–184.6 (184.0)	184.0–184.2 (184.1)	185.3–185.9 (185.6)
C—X	–	189.7–193.4 (191.6)	193.3–193.6 (193.4)	194.7

### 2.4 Thermal Behavior

**Scheme 2:**
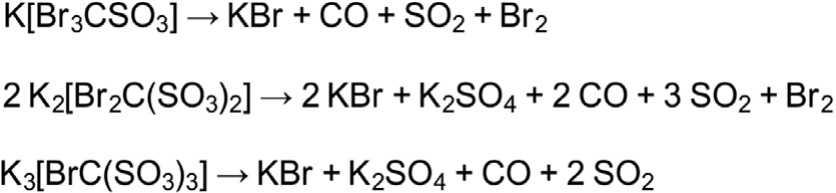
Decomposition reactions of the brominated methane sulfonates.

The three new bromomethanesulfonates are monohydrates. Upon heating, they all lose in a first step the crystal water in a temperature range between 60 and 120 °C. The thermal stability of the remaining anhydrous compounds increases with the number of sulfonate groups. Roughly, each sulfonate group leads to an increase of the decomposition temperature of 50 °C. Thus, K[Br_3_CSO_3_] decomposes with an onset temperature of about 250 °C, while for the disulfonate the respective temperature is 300 °C, and for K_3_[BrC(SO_3_)_3_] 350 °C. All decomposition reactions are exothermic. The residue of the decomposition of K[Br_3_CSO_3_] is KBr with respect to the X‐ray powder pattern. This is in line with the observed mass loss of 63.1%. The decomposition reaction can be found in **Scheme** **2**. For the disulfonate K_2_[Br_2_C(SO_3_)_2_], the residue of the decomposition is a mixture of KBr and K_2_SO_4_. This finding and the observed mass loss of 50% suggest a decomposition reaction according to Scheme 2 below. Also, the trisulfonate K_3_[BrC(SO_3_)_3_] gives KBr and K_2_SO_4_ as products of the thermal degradation (Scheme 2).

**Figure 5:**
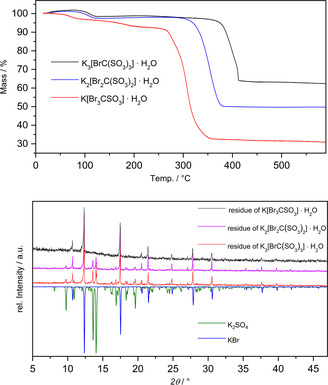
TG diagram of the decomposition of all three investigated potassium compounds and powder diffraction pattern of the decomposition residues (at bottom).

**Figure** **5** shows the curves of the thermogravimetric analyses (TG) of all three investigated potassium compounds and the powder pattern of the decomposition residues. Full data can be found in the supporting information.

### 2.5 Raman Spectroscopy

**Figure 6:**
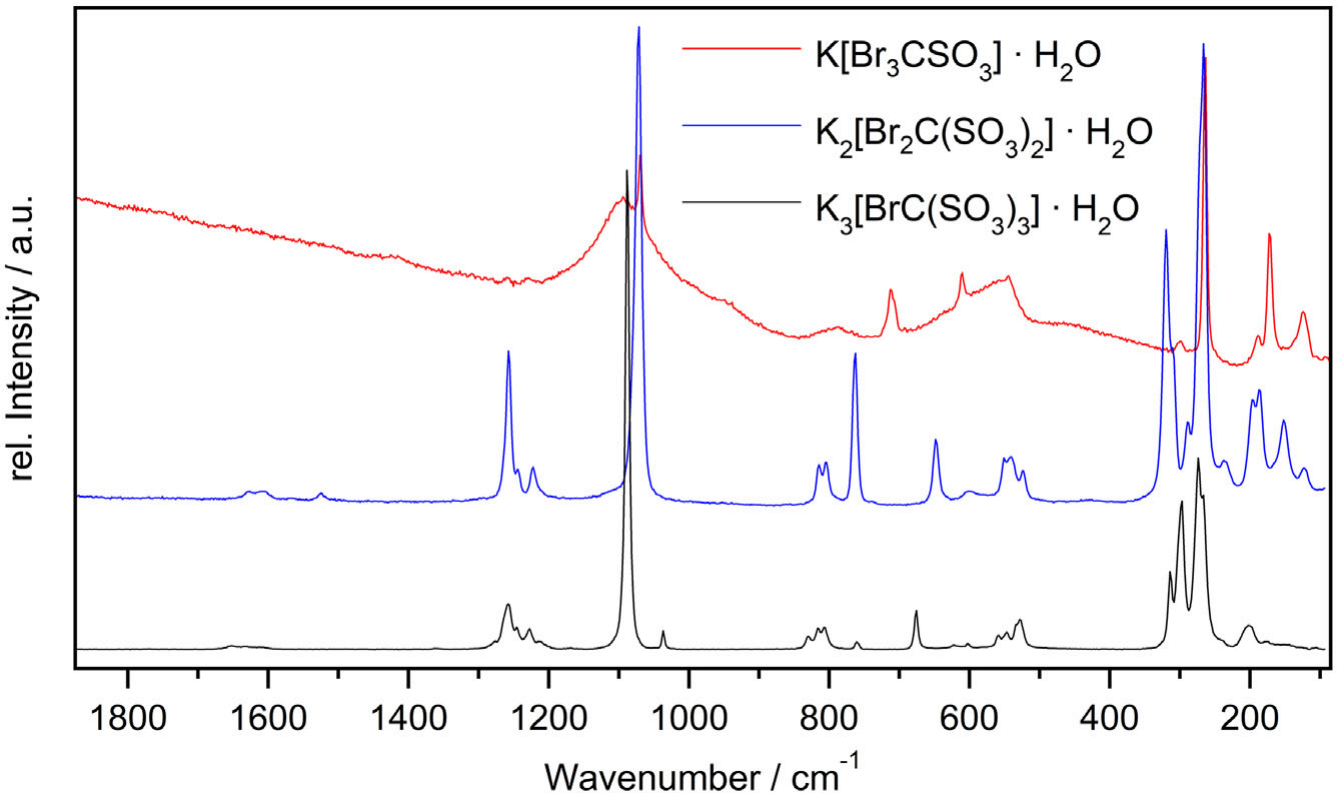
Raman spectrum of all three investigated potassium compounds. Comparison with theoretical data based on DFT calculations can be found in the Supporting Information.

The observed vibrations in the Raman spectra of the new bromomethane sulfonates could be assigned straightforwardly with respect to the DFT calculations of the anions. At higher energies the spectra are dominated by the stretching vibrations of the [SO_3_] groups which are found in a region from 1260 to 1222  cm^−^^1^ (*ν*_as_) and from 1093 to 1036 cm^−1^ (*ν*_s_), respectively. The vibration of the S—C bonds occurs between 788 and 759 cm^−1^. The lowest value is found for the [BrC(SO_3_)_3_]^3−^ anion, in accordance with the largest S—C bond for the three anions. The next transitions are located between 712 and 648 cm^−1^ and can be assigned to the C—Br stretching vibrations, followed by the deformation modes of the [SO_3_] groups, which range from 612 to 527 cm^−1^ (*δ*_as_ and *ν*_s_). At lower energies (288–151 cm^−1^) the deformation vibrations of the [CBr_*x*_] moieties are seen. **Figure** **6** shows the Raman spectrum of all three investigated potassium compounds; full data are shown in the supporting information.

## 3 Conclusion

In this contribution, we have introduced a simple and efficient way for the synthesis of brominated methanesulfonates. The anions [Br_3_CSO_3_]^−^, [Br_2_C(SO_3_)_2_]^2−^, and [BrC(SO_3_)_3_]^3−^ have been crystallized as their respective potassium salts, in all cases as monohydrates. The compounds have been comprehensively studied by thermal analyses and Raman spectroscopy, supported by DFT calculations. The thermal analyses show that all salts can be dehydrated. The anhydrous compounds are unknown up to now but would provide further interesting insights into the structure of the new anions. In any case, we are convinced that access to the new anions will lead to an intensive study of their coordination chemistry, and several new compounds will emerge from these studies. It will be interesting to compare the properties of these new materials with respect to the non‐halogenated derivatives and to their light halogen congeners. Finally, our findings encourage us to increase our efforts to prepare the final members of the methanesulfonate family, i.e., the respective iodine derivatives.

## Conflict of Interest

The authors declare no conflict of interest.

## Supporting information

Supplementary Material

## Data Availability

The data that support the findings of this study are available from the corresponding author upon reasonable request.
