# Supplementary material for: Brominated Methanesulfonates: Characterization of K[Br3CSO3] ⋅ H2O, K2[Br2C(SO3)2] ⋅ H2O and K3[BrC(SO3)3] ⋅ H2O
Source: ChemistryOpen. 2025 Nov 5;15(1):e202500455. doi: 10.1002/open.202500455 (PMC12831925; doi:10.1002/open.202500455)
Supplement: Supplementary file 1 — Supplementary Material [file OPEN-15-e202500455-s001.pdf]

Supporting Information

Brominated Methanesulfonates: Characterization of $K[Br_3CSO_3] \cdot H_2O$, $K_2[Br_2C(SO_3)_2] \cdot H_2O$ and $K_3[BrC(SO_3)_3] \cdot H_2O$

Katrin Eppers[a], Celina Sander[a] D. van Gerven[a] and Mathias S. Wickleder*[a]

Abstract:

Bromination reaction of aromatic methanesulfonates, such as phenyl methanesulfonate, $C_6H_5OSO_2CH_3$, with KOBr followed by hydrolytic cleavage of the aromatic ring generates potassium tribromomethanesulfonate “tribrate”, $K[Br_3CSO_3] \cdot H_2O$.[1-4] In contrast to the synthesis of the tribrate, in which an aromatic ring must be present at the SO_3 group for stabilization during bromination, the synthesis of potassium dibromomethanedisulfonate, $K_2[Br_2C(SO_3)_2] \cdot H_2O$ and potassium bromomethanetrisulfonate, $K_3[BrC(SO_3)_3] \cdot H_2O$ using KOBr works entirely without aromatic stabilization.

Comparing the C-S bond lengths of the different anions with each other, a trend emerges in which the C-S bond length increases with increasing number of SO_3 groups (decreasing number of Br atoms). Furthermore, an increase in thermal stability by approximately 50 °C per additional SO_3 group can be observed. The compounds were characterized by XRD, vibrational spectroscopy and thermal analyses.

Table of Contents

A Syntheses 2

$K[Br_3CSO_3] \cdot H_2O$ 2

$K_2[Br_2C(SO_3)_2] \cdot H_2O$ 2

$K_3[BrC(SO_3)_3] \cdot H_2O$ 3

B Structure determination and single crystal XRD data 3

$K[Br_3CSO_3] \cdot H_2O$ 3

$K_2[Br_2C(SO_3)_2] \cdot H_2O$ 6

$K_3[BrC(SO_3)_3] \cdot H_2O$ 9

Coordination sphere of the cations of the potassium methane(poly)sulfonates 12

C Powder XRD 13

$K_2[Br_2C(SO_3)_2] \cdot H_2O$ 13

$K_3[BrC(SO_3)_3] \cdot H_2O$ 14

D Thermal Analysis 15

$K[Br_3CSO_3] \cdot H_2O$ 15

$K_2[Br_2C(SO_3)_2] \cdot H_2O$ 17

$K_3[BrC(SO_3)_3] \cdot H_2O$ 18

E Vibrational Spectroscopy 19

$K[Br_3CSO_3] \cdot H_2O$ 19

$K_2[Br_2C(SO_3)_2] \cdot H_2O$ 19

$K_3[BrC(SO_3)_3] \cdot H_2O$ 20

F Results from Quantum Chemical Calculations 21

$K[Br_3CSO_3] \cdot H_2O$ 21

$K_2[Br_2C(SO_3)_2] \cdot H_2O$ 22

$K_3[BrC(SO_3)_3] \cdot H_2O$ 24

G References 25

A Syntheses

K[Br_3CSO_3] · H_2O

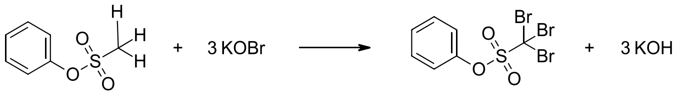

The first reaction step is the bromination of phenyl methanesulfonate using fresh KOBr solution. Therefore, 9.75 g KOH (174 mmol, 20 eq.) was dissolved in 22 ml H_2O, and the solution was cooled to 0 °C. Subsequently, 1.78 ml Br_2 (34.8 mmol, 4 eq.) was added dropwise resulting in the formation of a yellow KOBr solution. Meanwhile, 1.50 g phenyl methanesulfonate (8.70 mmol, 1 eq.) was dissolved in 33 ml dioxane. In the next step, the cooled KOBr solution was added dropwise to the solution of the starting material over a period of 10 min. Subsequently, the reaction mixture was stirred at 65 °C for 4 h, then cooled to room temperature. Et_2O and H_2O were added to the reaction mixture after which the organic phase was separated and washed with H_2O and brine. The organic phase was dried with $MgSO_4$, and the solvent was evaporated under reduced pressure. Approximately 2.45 g phenyl tribromomethanesulfonate (5.99 mmol) was isolated (69 %).

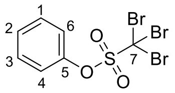

^{1}H-NMR (300 MHz, $CDCl_3$): δ [ppm] = 7.33 - 7.48 (5H, m, $CH_{arom.}$).
^{13}C-NMR (75 MHz, $CDCl_3$): δ [ppm] = 38.02 (C-7); 121.50 (C-4,-6); 127.93 (C-2); 130.11 (C-1,-3); 151.32 (C-5).

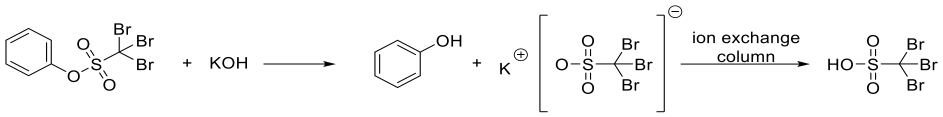

In a second step the aromatic ring was cleaved. 1.09 g KOH (19.5 mmol, 8 eq.) were dissolved in 10 ml H_2O and 1.0 g (2.44 mmol, 1 eq.) phenyl tribromomethanesulfonate was suspended in 7 ml 1,4-dioxane. To this solution, the previously prepared 10% KOH solution was added dropwise at room temperature. Subsequently, the reaction mixture was refluxed at 83 °C for 3 h, then cooled to room temperature. The solvent was evaporated under reduced pressure. The obtained yellow solid was again dissolved in a small amount of H_2O in order to obtain the acid replacing K^+ cations with H^+ cations using an ion exchange column. The solvent was again evaporated under reduced pressure and a yellow-orange oil was obtained, which was again dissolved in a small amount of H_2O and Et_2O. The organic layer was separated and the aqueous layer was extracted with Et_2O (3 x 20 ml). The solvent of the aqueous layer was again evaporated under reduced pressure and a red hygroscopic solid was obtained (0.61 g, 1.83 mmol, 75 %).

In order to obtain the potassium tribromomethanesulfonate 0.150 g (0.45 mmol, 1.0 eq.) of tribromomethanesulfonic acid was reacted with 0.044 g (0.72 mmol, 1.6 eq.) KOH in a small amount of water (1-2 ml). The reaction mixture was then heated to 80 °C until the water was evaporated to dryness. To the resulting residue acetonitrile was added, thus the tribrate dissolved, while the excess of KOH could be filtered off. The filtrate was finally left to stand in air so that acetonitrile could evaporate and crystals slowly formed. The obtained crystals of K[Br_3CSO_3] · H_2O were colorless and elongated in one direction.

$K_2[Br_2C(SO_3)_2]$ · H_2O

$$Na_2[H_2C(SO_3)_2]\cdot H_2O + 2\,KOBr \longrightarrow K_2[Br_2C(SO_3)_2]\cdot H_2O + 2\,NaOH$$

Potassium hydroxide (2.569 g, 45.79 mmol, 8.0 eq.) was dissolved in water (25 ml) and cooled down to 0 °C. To this solution 0.59 ml bromine (11.51 mmol, 2.0 eq.) was added slowly to obtain the yellow potassium hypobromite solution. Commercially available (BLDpharm) sodium methanedisulfonate (1.259 g, 5.72 mmol, 1.0 eq.) was dissolved in water (10 ml) and the KOBr solution was added in portions. During this process, the solution turned dark-red and in the end yellow again. The mixture was heated to 70 °C for 4 h. The solution was then concentrated under reduced pressure and cooled to obtain a colourless solid, that was filtered off. The remaining solution was again concentrated and cooled to repeat this process. The combined product was obtained as a colourless solid (1.11 g, 2.71 mmol, 47 %). Single crystals for SC-XRD measurements were obtained by recrystallization from water and identified as $K_2[Br_2C(SO_3)_2]$ · H_2O.

$K_3[BrC(SO_3)_3] \cdot H_2O$

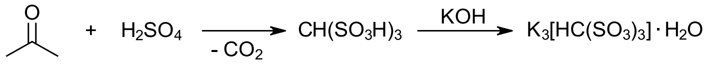

Potassium methanetrisulfonate was obtained by reacting 10 mL (127 mmol) of oleum (65% SO_3 content) with acetone under argon protective gas at a temperature of roughly -10 °C. 1.75 mL (23.8 mmol) of cooled acetone was added dropwise to the cooled Oleum, with the temperature remaining between -10 °C and -3 °C. The reaction mixture was then heated to 80 °C for 1.5 hours. allowed to cool, and poured onto ice water. Finally, it was neutralized with KOH. The solid obtained after neutralization was recrystallized several times in aqueous solution to separate the $K_3[HC(SO_3)_3]{\cdot}H_2O$ from the byproduct K_2SO_4.

$$K_3[HC(SO_3)_3] \cdot H_2O \;+\; KOBr \longrightarrow K_3[BrC(SO_3)_3] \cdot H_2O \;+\; KOH$$

Potassium hydroxide (0.964 g, 17.12 mmol, 4.0 eq.) was dissolved in water (10 ml) and cooled down to 0 °C. To this solution 0.22 ml bromine (4.28 mmol, 1.0 eq.) was added slowly to obtain the yellow potassium hypobromite solution. Potassium methanetrisulfonate (2.00 g, 4.28 mmol, 1.0 eq.) was dissolved in 70 ml warm water (45 °C) and the KOBr solution was added slowly. The mixture was heated to 75 °C for 6 h. Stirring at room temperature over night with subsequent cooling at 0 °C led to a colourless solid, that was filtered off. The product was obtained as a colourless solid (1.50 g, 3.33 mmol, 78 %), which could be identified as $K_3[BrC(SO_3)_3] \cdot H_2O$.

B Structure determination and single crystal XRD data

Single crystal structure determination has been performed on a Bruker D8 VENTURE KAPPA diffractometer with a microfocus sealed tube using a multilayer mirror as monochromator and a Bruker PHOTON III detector. MoK_{α} radiation (71.073 pm) was used as X-ray source. The crystals were prepared in perfluorinated ether (Fomblin® YR-180) and selected with the aid of a light microscope with a polarization filter. The crystals were fixed on a micromount with a 150 µm polymer loop and adjusted to the X-ray beam under cooling at 100 K. The intensity data were collected and the images processed using APEX4. The integration was done with SAINT and a multi-scan absorption correction using SADABS was applied.[5-6] The structure solution was performed in the software Olex2 by intrinsic phasing (SHELXT) and the structural model was refined by least squares methods using SHELXL.[7-8] For $K_3[BrC(SO_3)_3]{\cdot}H_2O$ the hydrogen atoms of the H_2O molecule have been refined without restrictions. The hydrogen atoms in $K_2[Br_2C(SO_3)_2]{\cdot}H_2O$ and $K[Br_3CSO_3]{\cdot}H_2O$ were treated according to a riding model.

$K[Br_3CSO_3] \cdot H_2O$

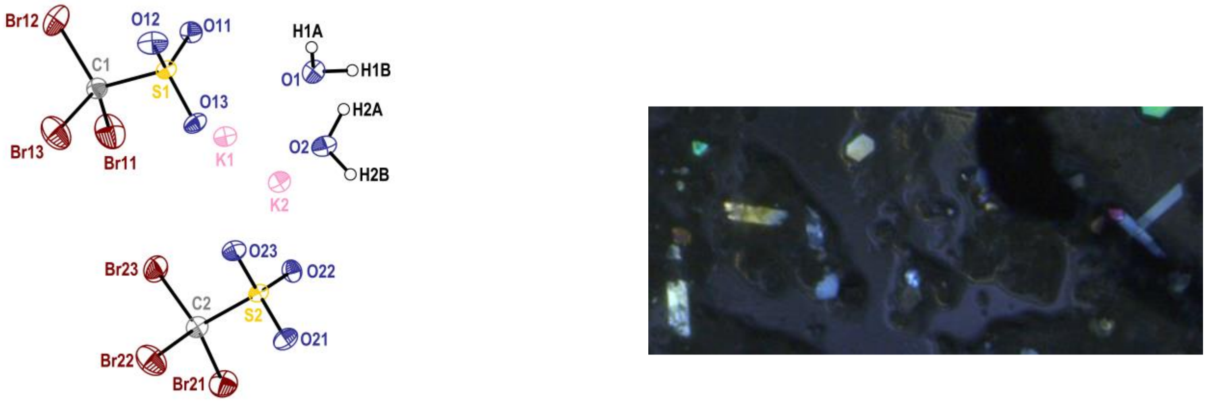

Figure S1: Structure and labelling of **$K[Br_3CSO_3] \cdot H_2O$** (left) and light microscope image of the single crystals (right).

Table S1: Crystal data and structure refinement for **$K[Br_3CSO_3] \cdot H_2O$**.

Empirical formula	$K[Br_3CSO_3] \cdot H_2O$
Formula weight	388.92 g · mol^{-1}
Temperature	100.0(2) K
Wavelength	71.073 pm (Mo-K$_\alpha$)
Crystal system	triclinic
Space group	$P\bar{1}$ (2)
Unit cell dimensions	a = 662.87(4) pm
	b = 1090.98(7) pm
	c = 1273.98(8) pm
	α = 106.079(2)°
	β = 93.438(2)°
	γ = 90.121(2)°
Volume	0.8835(1) nm^3
Z	4
Density (calculated)	2.924 g · cm^{-3}
Absorption coefficient μ	14.363 mm^{-1}
F(000)	720
Crystal size	0.02 × 0.01 × 0.01 mm^3
2θ range for data collection	3.886 to 59.998
Index ranges	$-9 \le h \le 9$, $-15 \le k \le 15$, $-17 \le l \le 17$
Reflections collected	66415
Independent reflections	5150 [R_{int}= 0.0563, R_σ= 0.0265]
Absorption correction	multiscan
Max. and min. transmission	0.7474 and 0.5357
Refinement method	least-squares
Data / restraints / parameters	5150/0/197
Goodness-of-fit on F^2	1.095
Final R indices [$I \ge 2\sigma(I)$]	R_1 = 0.0421, wR_2 = 0.1106
R indices (all data)	R_1 = 0.0543, wR_2 = 0.1186
Largest diff. peak and hole	1.61 /−1.66 e · Å^{-3}
CCDC number	2386367

Table S2: Atomic coordinates and equivalent isotropic displacement parameters [pm^2 x 10^4] for **$K[Br_3CSO_3] \cdot H_2O$**. *U(eq)* is defined as one third of the trace of the orthogonalized U_{ij} tensor.

Atom	*x*	*y*	*z*	U_{eq}
Br23	0.84271(8)	0.50948(5)	0.14707(5)	0.0247(1)
Br21	0.75552(9)	0.23153(5)	0.16463(5)	0.0268(1)
Br11	1.3168(1)	0.74890(6)	0.14920(5)	0.0349(2)
Br12	1.2772(1)	1.04021(7)	0.16553(6)	0.0360(2)
Br22	0.40146(9)	0.39121(6)	0.09813(5)	0.0319(2)
Br13	0.8964(1)	0.85710(7)	0.09748(6)	0.0374(2)
K1	0.6318(1)	0.84216(9)	0.41697(9)	0.0145(2)
K2	1.1349(1)	0.6052(1)	0.41590(9)	0.0148(2)
S1	1.0892(2)	0.9228(1)	0.33024(9)	0.0118(2)
S2	0.5950(2)	0.4674(1)	0.32951(9)	0.0115(2)
O22	0.7873(5)	0.4652(3)	0.3888(3)	0.0156(6)
O11	1.2821(5)	0.9604(3)	0.3895(3)	0.0159(6)
O13	1.0086(5)	0.8032(3)	0.3380(3)	0.0172(7)
O2	0.8614(5)	0.7325(3)	0.5567(3)	0.0171(7)
O1	1.3621(5)	0.8036(3)	0.5572(3)	0.0168(7)
O21	0.4424(5)	0.3819(3)	0.3487(3)	0.0177(7)
O23	0.5202(5)	0.5932(3)	0.3352(3)	0.0164(7)
O12	0.9412(5)	1.0234(3)	0.3477(3)	0.0205(7)
C1	1.1441(7)	0.8931(5)	0.1859(4)	0.0156(8)
C2	0.6472(7)	0.3991(4)	0.1844(4)	0.0147(8)

Table S3: Anisotropic displacement parameters [pm^2 x 10^4] for **$K[Br_3CSO_3] \cdot H_2O$**. The anisotropic displacement factor exponent takes the form: $-2\pi^2$ [$h^2 a^{*2} U_{11}$ + 2 h k a* b* U_{12} +…].

Atom	U_{11}	U_{22}	U_{33}	U_{23}	U_{13}	U_{12}
Br23	0.0241(3)	0.0286(3)	0.0242(3)	0.0111(2)	0.0043(2)	-0.0049(2)
Br21	0.0333(3)	0.0182(2)	0.0287(3)	0.0043(2)	0.0082(2)	0.0095(2)
Br11	0.0381(3)	0.0363(3)	0.0278(3)	0.0030(2)	0.0089(3)	0.00169(3)

Br12	0.0460(4)	0.0344(3)	0.0345(3)	0.0201(3)	0.0058(3)	-0.0081(3)
Br22	0.0265(3)	0.0365(3)	0.0283(3)	0.0031(2)	-.00050(2)	0.0032(2)
Br13	0.0327(3)	0.0465(4)	0.0300(3)	0.0085(3)	-0.0116(3)	-0.0010(3)
K1	0.0104(4)	0.0136(4)	0.0192(5)	0.0035(3)	0.0030(3)	0.0017(3)
K2	0.0101(4)	0.0161(4)	0.0188(5)	0.0054(4)	0.0025(3)	0.0006(3)
S1	0.0098(4)	0.0105(4)	0.0154(5)	0.0037(4)	0.0029(4)	0.0021(3)
S2	0.0098(4)	0.0103(4)	0.0150(5)	0.0038(4)	0.0028(4)	0.0010(3)
O22	0.012(2)	0.019(2)	0.014(2)	0.005(1)	-0.001(1)	0.000(1)
O11	0.012(2)	0.017(2)	0.018(2)	0.004(1)	-0.001(1)	0.000(1)
O13	0.017(2)	0.015(2)	0.021(2)	0.007(1)	0.004(1)	-0.002(1)
O2	0.014(2)	0.014(2)	0.022(2)	0.004(1)	0.005(1)	0.000(1)
O1	0.014(2)	0.017(2)	0.020(2)	0.006(1)	0.003(1)	0.004(1)
O21	0.012(2)	0.017(2)	0.024(2)	0.008(1)	0.004(1)	-0.001(1)
O23	0.017(2)	0.010(2)	0.020(2)	0.003(1)	0.003(1)	0.005(1)
O12	0.013(2)	0.014(2)	0.032(2)	0.002(1)	0.004(1)	0.007(1)
C1	0.015(2)	0.015(2)	0.017(2)	0.005(2)	0.000(2)	0.001(2)
C2	0.011(2)	0.015(2)	0.018(2)	0.004(2)	0.003(2)	0.002(2)

Table S4: Experimental bond lengths [pm] for **K[Br_3CSO_3] · H_2O**.

Atom–Atom	Length	Atom–Atom	Length	Atom–Atom	Length
Br23–C2	193.4(5)	K1–O23	271.0(4)	S1–O13	143.9(3)
Br21–C2	192.4(5)	K1–O12	317.2(4)	S1–C1	183.5(5)
Br11–C1	191.5(5)	K2–O1	277.4(4)	S2–O22	144.6(3)
Br12–C1	191.7(5)	K2–O21[1]	314.6(4)	S2–O21	145.1(4)
Br22–C2	189.7(5)	K2–O23[1]	279.9(4)	S2–O23	144.4(3)
Br13–C1	191.0(5)	K2–O22[5]	282.0(4)	S2–C2	184.6(5)
K1–O11[4]	281.6(4)	K2–O22	271.4(4)		
K1–O11[3]	270.7(3)	K2–O13	272.9(4)		
K1–O13	273.9(4)	K2–O2	273.0(4)		
K1–O2	278.9(5)	S1–O11	144.3(4)		
K1–O1[3]	271.4(4)	S1–O12	145.3(3)		

Symmetry transformations used to generate equivalent atoms: [1]1+X,+Y,+Z; [2]1-X,2-Y,1-Z; [3]-1+X,+Y,+Z; [4]2-X,2-Y,1-Z; [5]2-X,1-Y,1-Z.

Table S5: Experimental bond angles [°] for the anion in **K[Br_3CSO_3] · H_2O**.

Atom–Atom–Atom	Angle [°]	Atom–Atom–Atom	Angle [°]
O11–S1–C1	104.3(2)	S2–C2–Br23	108.0(2)
O13–S1–O11	114.5(2)	Br11–C1–Br12	110.0(2)
O13–S1–O12	113.2(2)	Br13–C1–Br11	110.0(2)
O13–S1–C1	104.6(2)	Br13–C1–Br12	110.4(2)
O12–S1–C1	104.3(2)	S1–C1–Br11	107.9(2)
O22–S2–O21	114.1(2)	S1–C1–Br12	109.5(2)
O22–S2–C2	104.5(2)	S1–C1–Br13	109.1(2)
O21–S2–C2	104.1(2)	Br21–C2–Br23	109.9(2)
O23–S2–O22	115.1(2)	Br22–C2–Br23	110.6(2)
O23–S2–O21	113.1(2)	S2–C2–Br21	109.4(2)
O23–S2–C2	104.5(2)	S2–C2–Br22	108.3(2)
Br22–C2–Br21	110.6(2)	O23–S2–O22	115.1(2)

Symmetry transformations used to generate equivalent atoms: [1]1+X,+Y,+Z; [2]-1+X,+Y,+Z; [3]1-X,2-Y,1-Z; [4]2-X,2-Y,1-Z; [5]2-X,1-Y,1-Z.

Table S6: Experimental torsion angles [°] for the anion in **K[Br_3CSO_3] · H_2O**.

Atom–Atom–Atom–Atom	Torsion Angle [°]	Atom–Atom–Atom–Atom	Torsion Angle [°]
O22–S2–C2–Br23	−62.2(3)	O21–S2–C2–Br23	177.8(2)
O22–S2–C2–Br21	57.3(3)	O21–S2–C2–Br22	57.9(3)
O22–S2–C2–Br22	177.9(2)	O21–S2–C2–Br21	−62.7(3)
O11–S1–C1–Br11	65.8(3)	O23–S2–C2–Br23	59.0(3)
O11–S1–C1–Br12	−54.9(3)	O23–S2–C2–Br21	178.5(2)
O11–S1–C1–Br13	−174.8(2)	O23–S2–C2–Br22	−60.9(3)
O13–S1–C1–Br11	−54.8(3)	O12–S1–C1–Br11	−173.9(2)
O13–S1–C1–Br12	−174.5(2)	O12–S1–C1–Br12	66.4(3)
O13–S1–C1–Br13	64.6(3)	O12–S1–C1–Br13	−54.5(3)

Symmetry transformations used to generate equivalent atoms: [1]-1+X,+Y,+Z; [2]2-X,1-Y,1-Z; [3]2-X,2-Y,1-Z; [4]1+X,+Y,+Z.

Table S7: Hydrogen bonds in **K[Br$_3$CSO$_3$] · H$_2$O**: Observed donor–acceptor distances (O–O) [pm] and angles (O–H…O) [°].

Donor–Acceptor	Length/	Angle (O-H...O)
O1–O12	284.9(5)	157.6(2)
O1–O21	289.5(5)	139.4(2)
O2–O12	287.8(4)	139.1(2)
O2–O21	286(5)	157.4(2)

K$_2$[Br$_2$C(SO$_3$)$_2$] · H$_2$O

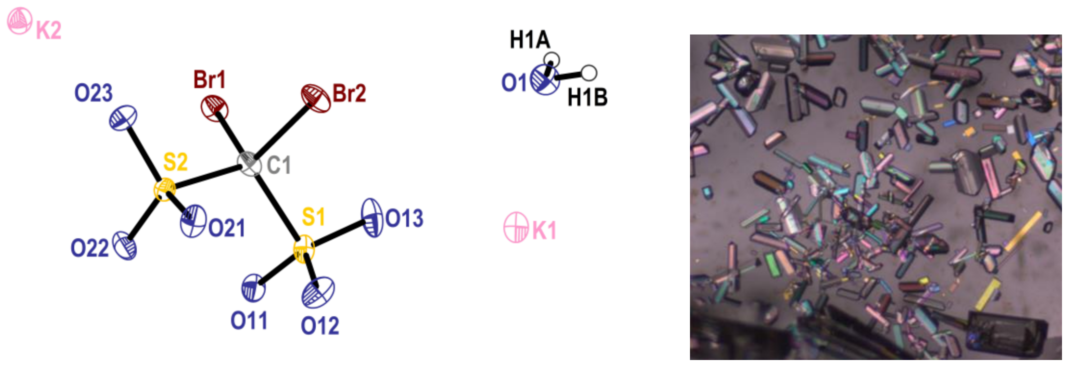

Figure S2: Structure and labelling of **K$_2$[Br$_2$C(SO$_3$)$_2$] · H$_2$O** (left) and light microscope image of the single crystals (right).

Table S8: Crystal data and structure refinement for **K$_2$[Br$_2$C(SO$_3$)$_2$] · H$_2$O**.

Empirical formula	$CH_2Br_2K_2O_7S_2$
Formula weight	427.68 g · mol^{-1}
Temperature	100.0(2) K
Wavelength	71.073 pm (Mo-K$_\alpha$)
Crystal system	monoclinic
Space group	$P2_1/c$ (14)
Unit cell dimensions	a = 717.17(3) pm
	b = 710.78(3) pm
	c = 2092.50(9) pm
	β = 94.732(2)°
Volume	1.06302(8) nm^3
Z	4
Density (calculated)	2.672 g · cm^{-3}
Absorption coefficient μ	8.802 mm^{-1}
F(000)	815
Crystal size	0.34 × 0.09 × 0.04 mm^3
2θ range for data collection	3.906 to 56.572
Index ranges	$-8 \le h \le 9$, $-9 \le k \le 9$, $-27 \le l \le 27$
Reflections collected	17399
Independent reflections	2616 [R_{int}= 0.0442, R_σ= 0.0288]
Absorption correction	multiscan
Max. and min. transmission	0.7457 and 0.4215
Refinement method	least-squares
Data / restraints / parameters	2616/0/129
Goodness-of-fit on F^2	1.186
Final R indices [$I \ge 2\sigma(I)$]	R_1 = 0.0260, wR_2 = 0.0641
R indices (all data)	R_1 = 0.0276, wR_2 = 0.0649
Largest diff. peak and hole	0.77/-1.00 e · Å^{-3}
CCDC number	2341531

Table S9: Atomic coordinates and equivalent isotropic displacement parameters [pm^2 x 10^4] for $\mathbf{K_2[Br_2C(SO_3)_2] \cdot H_2O}$. *U(eq)* is defined as one third of the trace of the orthogonalized U_{ij} tensor**.**

Atom	*x*	*y*	*z*	U_{eq}
Br1	0.52557(3)	0.44717(3)	0.37226(2)	0.01277(9)
Br2	0.58955(3)	0.81078(3)	0.29007(2)	0.01545(9)
K2	0.17574(6)	0.70053(6)	0.49086(2)	0.0128(1)
K1	1.03958(7)	0.61231(6)	0.18081(2)	0.0135(1)
S2	0.68048(7)	0.80881(7)	0.43601(3)	0.0094(1)
S1	0.92224(7)	0.57858(7)	0.35022(3)	0.0109(1)
O23	0.4833(2)	0.8358(2)	0.44379(8)	0.0139(3)
O1	0.7226(2)	0.6459(2)	0.10945(8)	0.0164(3)
O22	0.7756(2)	0.6932(2)	0.48593(8)	0.0145(3)
O11	0.9695(2)	0.4477(2)	0.40233(8)	0.0129(3)
O13	0.9020(2)	0.4873(2)	0.28810(8)	0.0182(3)
O12	1.0368(2)	0.7461(2)	0.35325(9)	0.0171(3)
O21	0.7790(2)	0.9814(2)	0.42393(8)	0.0155(3)
C1	0.6850(3)	0.6632(3)	0.3633(1)	0.0110(4)

Table S10: Anisotropic displacement parameters [pm^2 × 10^4] for $\mathbf{K_2[Br_2C(SO_3)_2] \cdot H_2O}$. The anisotropic displacement factor exponent takes the form: $-2\pi^2 [\, h^2 a^{*2} U_{11} + 2 h k a^* b^* U_{12} + \ldots]$.

Atom	U_{11}	U_{22}	U_{33}	U_{23}	U_{13}	U_{12}
Br1	0.0152(1)	0.0104(1)	0.0124(1)	-0.00015(7)	-0.00081(9)	-0.00359(7)
Br2	0.0197(1)	0.0140(1)	0.0118(1)	0.0041(8)	-0.00378(9)	0.00001(8)
K2	0.0133(2)	0.0127(2)	0.0126(2)	0.0003(2)	0.00232(18)	-0.0024(2)
K1	0.0178(2)	0.0100(2)	0.0132(2)	0.0003(2)	0.00368(18)	0.0007(2)
S2	0.0101(2)	0.0086(2)	0.0096(2)	-0.0008(2)	0.00170(19)	-0.0000(2)
S1	0.0151(2)	0.0083(2)	0.0099(3)	0.0008(2)	0.0037(2)	0.0015(2)
O23	0.0105(7)	0.0146(8)	0.0170(8)	-0.0002(6)	0.0031(6)	0.0018(6)
O1	0.0189(8)	0.0133(7)	0.0169(8)	0.0028(7)	0.0014(6)	-0.0032(6)
O22	0.0182(8)	0.0162(8)	0.0087(8)	-0.0003(6)	-0.0005(6)	0.0037(6)
O11	0.0149(7)	0.0122(8)	0.0119(8)	0.0020(6)	0.0030(6)	0.0033(5)
O13	0.0305(9)	0.0147(8)	0.0101(8)	-0.001(6)	0.0062(7)	0.0048(7)
O12	0.0154(7)	0.0108(8)	0.0259(9)	0.0024(7)	0.0064(7)	-0.0009(6)
O21	0.0187(8)	0.0120(7)	0.0164(8)	-0.0037(6)	0.0058(7)	-0.0055(6)
C1	0.0135(9)	0.0093(9)	0.010(1)	0.0004(8)	-0.0007(8)	-0.0016(7)

Table S11: Experimental bond lengths [pm] for $\mathbf{K_2[Br_2C(SO_3)_2] \cdot H_2O}$.

Atom–Atom	Length	Atom–Atom	Length
Br1–K2^1	358.92(6)	K1–O13	267.8(2)
Br1–C1	193.3(2)	K1–O13^2	276.7(2)
Br2–K1^2	343.10(5)	K1–O12^7	274.2(2)
Br2–C1	193.6(2)	K1–O21^7	279.9(2)
K2–O23	266.8(2)	S2–O23	145(2)
K2–O22^5	286.4(2)	S2–O22	145.4(2)
K2–O22^1	285.7(2)	S2–O21	144.8(2)
K2–O11^1	275.2(2)	S2–C1	184.2(2)
K2–O11^5	289.8(2)	S1_O11	145.2(2)
K2–O12^5	298.6(2)	S1–O13	144.9(2)
K2–O21^6	288.1(2)	S1–O12	144.5(2)
K1–O1	262.6(2)	S1–C1	184(2)
K1–O11^2	294.9(2)		

Symmetry transformations used to generate equivalent atoms: ^{1}1-X,1-Y,1-Z; ^{2}2-X,1/2+Y,1/2-Z; ^{3}-X,1-Y,1-Z; ^{4}-1+X,3/2-Y,1/2+Z; ^{5}-1+X,+Y,+Z; ^{6}1-X,2-Y,1-Z; ^{7}2-X,-1/2+Y,1/2-Z

Table S12: Experimental bond angles [°] for the anion in $\mathbf{K_2[Br_2C(SO_3)_2] \cdot H_2O}$.

Atom–Atom–Atom	Angle [°]
O23–S2–K2[9]	155.20(7)
O23–S2–O22	113.5(1)
O23–S2–C1	104.5(1)
O22–S2–C1	103.66(9
O21–S2–O23	113.7(1)
O21–S2–O22	113.4(1)
O21–S2–C1	106.85(9)
O11–S1–C1	104.9(1)
O13–S1–O11	112.7(1)
O13–S1–C1	104.7(1)
O12–S1–O11	113.5(1)
O12–S1–O13	115.0(1)
O12–S1–C1	104.65(9)
Br1–C1–Br2	109.5(1)
S2–C1–Br1	108.4(1)
S2–C1–Br2	108.7(1)
S2–C1–S1	112.7(1)
S1–C1–Br1	108.4(1)
S1–C1–Br2	109.2(1)

Table S13: Selected experimental torsion angles [°] for the anion in $\mathbf{K_2[Br_2C(SO_3)_2] \cdot H_2O}$.

Atom–Atom–Atom–Atom	Torsion Angle [°]
O23–S2–C1–Br1	−51.2(1)
O23–S2–C1–Br2	67.7(1)
O23–S2–C1–S1	−171.1(1)
O22–S2–C1–Br1	67.9(1)
O22–S2–C1–Br2	−173.2(1)
O22–S2–C1–S1	−52.0(1)
O11–S1–C1–Br1	−52.4(1)
O11–S1–C1–Br2	−171.6(1)
O11–S1–C1–S2	67.6 (1)
O13–S1–C1–Br1	66.5(1)
O13–S1–C1–Br2	−52.7(1)
O13–S1–C1–S2	−173.5(1)
O12–S1–C1–Br1	−172.1(1)
O12–S1–C1–Br2	68.7(1)
O12–S1–C1–S2	−52.2(1)
O21–S2–C1–Br1	−172.0(1)
O21–S2–C1–Br2	−53.1(1)
O21–S2–C1–S1	68.0(1)

Table S14: Hydrogen bonds in $\mathbf{K_2[Br_2C(SO_3)_2] \cdot H_2O}$: Observed donor–acceptor distances (O–O) [pm] and angles (O–H…O) [°].

Donor–Acceptor	Length/	Angle (O-H...O)
O1–O22	288.0(2)	175.4(2)
O1–O23	282.9(1)	159.7(2)

$K_3[BrC(SO_3)_3] \cdot H_2O$

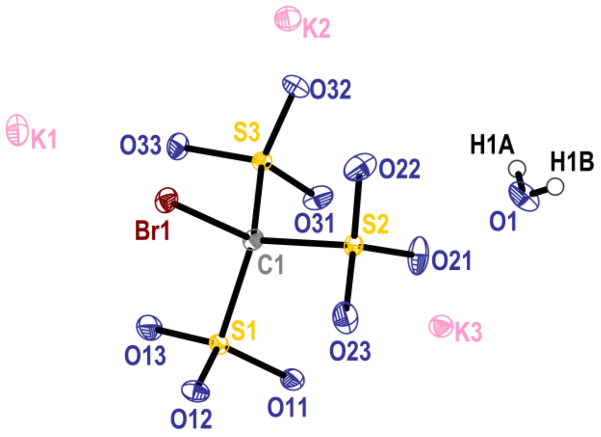

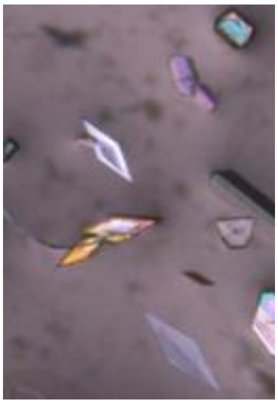

Figure S3: Structure and labelling of **$K_3[BrC(SO_3)_3] \cdot H_2O$** (left) and light microscope image of the single crystals (right).

Table S15: Crystal data and structure refinement for **$K_3[BrC(SO_3)_3] \cdot H_2O$**.

Empirical formula	$CH_2BrK_3O_{10}S_3$
Formula weight	467.42 g · mol^{-1}
Temperature	100.0(2) K
Wavelength	71.073 pm (Mo-K$_\alpha$)
Crystal system	tetragonal
Space group	$P4_3$ (78)
Unit cell dimensions	a = 713.6(1) pm
	b = 713.6(1) pm
	c = 2324.50(7) pm
Volume	1.18379(5) nm^3
Z	4
Density (calculated)	2.623 g · cm^{-3}
Absorption coefficient μ	5.097 mm^{-1}
F(000)	912
Crystal size	0.01 × 0.01 × 0.03 mm^3
2θ range for data collection	5.71 to 52.73
Index ranges	$-8 \leq h \leq 8$, $-8 \leq k \leq 8$, $-29 \leq l \leq 29$
Reflections collected	43567
Independent reflections	2418 [R_{int}= 0.0584, R_σ= 0.0291]
Completeness to theta = 25.242°	100.0%
Absorption correction	multiscan
Max. and min. transmission	0.7478 and 0.5987
Refinement method	least-squares
Data / restraints / parameters	2418 / 1 / 171
Goodness-of-fit on F^2	1.153
Final R indices [$I \geq 2\sigma(I)$]	R_1 = 0.0166, wR_2 = 0.0384
R indices (all data)	R_1 = 0.0170, wR_2 = 0.0385
Largest diff. peak and hole	0.26 /– 0-67 e · Å^{-3}
Flack parameter	0.001(3)
CCDC number	2341533

Table S16: Atomic coordinates and equivalent isotropic displacement parameters [pm^2 x 10^4] for **$K_3[BrC(SO_3)_3] \cdot H_2O$**. *U(eq)* is defined as one third of the trace of the orthogonalized U_{ij} tensor**.**

Atom	*x*	*y*	*z*	U_{eq}
Br1	0.27710(5)	1.22583(5)	0.49329(2)	0.0095(1)
K3	0.8334(1)	0.6105(1)	0.54775(3)	0.0089(2)
K2	0.5874(1)	1.3912(1)	0.38707(3)	0.0111(2)
K1	−0.0577(1)	0.9390(1)	0.40957(3)	0.0123(2)
S2	0.6805(1)	1.1316(1)	0.52951(3)	0.0067(2)
S3	0.4783(1)	0.8845(1)	0.44292(4)	0.0061(2)
S1	0.3506(1)	0.8822(1)	0.56913(3)	0.0067(2)
O33	0.2915(3)	0.8389(4)	0.4231(1)	0.0101(5)
O32	0.5733(3)	1.0095(3)	0.4029(1)	0.0100(5)
O22	0.7161(4)	1.2741(4)	0.4867(1)	0.0163(6)
O21	0.8145(4)	0.9803(4)	0.5281(1)	0.0134(5)
O12	0.2975(3)	1.0207(3)	0.6126(1)	0.0102(5)
O31	0.5881(4)	0.7222(4)	0.4586(1)	0.0111(5)
O13	0.1937(4)	0.7837(4)	0.5446(1)	0.0124(5)
O1	1.0371(4)	0.5521(5)	0.4566(1)	0.0135(6)
O11	0.5012(4)	0.7606(4)	0.5885(1)	0.0105(5)
O23	0.6536(4)	1.2092(4)	0.5866(1)	0.0154(6)
C1	0.4523(5)	1.0236(5)	0.5097(1)	0.0068(7)
H1A	1.023(6)	0.486(7)	0.431(2)	0.01(1)
H1B	1.111(8)	0.603(8)	0.456(2)	0.02(2)

Table S17: Anisotropic displacement parameters [$pm^2 \times 10^4$] for **$K_3[BrC(SO_3)_3] \cdot H_2O$**. The anisotropic displacement factor exponent takes the form: $-2\pi^2$ [$h^2 a^{*2} U_{11}$ + 2 h k a* b* U_{12} +…].

Atom	U_{11}	U_{22}	U_{33}	U_{23}	U_{13}	U_{12}
Br1	0.0091(2)	0.0089(2)	0.0105(2)	0.0003(1)	−0.0006(1)	0.0029(1)
K3	0.0097(4)	0.0092(4)	0.0079(3)	0.0001(3)	0.0005(3)	0.0012(3)
K2	0.0117(4)	0.0097(4)	0.0119(3)	0.0034(3)	0.0002(3)	0.0002(3)
K1	0.0080(4)	0.0119(4)	0.0171(4)	0.0010(3)	0.0001(3)	0.0005(3)
S2	0.0060(4)	0.0064(4)	0.0077(4)	−0.0004(3)	−0.0007(3)	−0.0003(3)
S3	0.0065(4)	0.0064(4)	0.0054(3)	−0.0008(3)	−0.0002(3)	0.0002(3)
S1	0.0061(4)	0.0074(4)	0.0066(4)	0.0002(3)	0.0010(3)	0.0003(3)
O33	0.008(1)	0.012(1)	0.011(1)	−0.002(1)	−0.0021(9)	−0.003(1)
O32	0.011(1)	0.010(1)	0.009(1)	−0.0001(9)	0.004 (1)	−0.001(1)
O22	0.018(1)	0.015(1)	0.02(2)	0.008(1)	−0.007(1)	−0.011(1)
O21	0.008(1)	0.008(1)	0.024(1)	−0.002(1)	−0.002(1)	0.0030(1)
O12	0.013(1)	0.011(1)	0.007(1)	−0.0012(9)	0.002(1)	0.001(1)
O31	0.016(1)	0.008(1)	0.009(1)	−0.001(1)	−0.001(1)	0.005(1)
O13	0.010(1)	0.016(1)	0.011(1)	0.0007(1)	−0.000(1)	−0.006(1)
O1	0.014(2)	0.015(2)	0.013(1)	−0.006(1)	0.004(1)	−0.007(1)
O11	0.01(1)	0.013(1)	0.009(1)	0.0035(9)	0.0019(9)	0.004(1)
O23	0.010(1)	0.024(2)	0.012(1)	−0.008(1)	−0.000(1)	−0.001(1)
C1	0.007(2)	0.006(2)	0.007(2)	−0.000(1)	−0.000(1)	0.002(1)

Table S18: Experimental bond lengths [pm] for the anion in **$K_3[BrC(SO_3)_3] \cdot H_2O$.**

Atom–Atom	Length	Atom–Atom	Length
Br1–C1	194.7(3)	S3–C1	185.3(3)
S2–O22	144.5(3)	S1–O12	146.3(3)
S2–O21	144.3(3)	S1–O13	144.0(3)
S2–O23	145.0(3)	S1–O11	145.3(3)
S2–C1	185.9(4)	S1–C1	185.7(3)
S3–O33	144.8(3)	O1–H1A	76.0(5)
S3–O32	145.6(3)	O1–H1B	64.0(5)
S3–O31	144.5(3)		

Table S19: Selected experimental bond angles [°] for the anion in **$K_3[BrC(SO_3)_3] \cdot H_2O$**.

Atom–Atom–Atom	Angle [°]
O22–S2–C1	106.0(2)
O22–S2–O23	112.6(2)
O21–S2–O22	113.2(2)
O21–S2–O23	113.2(2)
O21–S2–C1	105.4(2)
O23–S2–C1	105.6(2)
O33–S3–O32	111.3(2)
O33–S3–C1	107.2(2)
O32–S3–C1	104.7(2)
O31–S3–O33	113.6(2)
O31–S3–O32	113.5(2)
O31–S3–C1	105.8(2)
O12–S1–C1	104.3(2)
O13–S1–O12	113.7(2)
O13–S1–O11	113.9(2)
O13–S1–C1	105.9(2)
O11–S1–O12	112.4(2)
O11–S1–C1	105.4(2)
H1A–O1–H1B	117(6)
S2–C1–Br1	107.7(2)
S3–C1–Br1	107.3(2)
S3–C1–S2	110.0(2)
S3–C1–S1	111.8(2)
S1–C1–Br1	107.3(2)
S1–C1–S2	112.5(2)

Table S20: Selected experimental torsion angles **[°]** for the anion in **$K_3[BrC(SO_3)_3] \cdot H_2O$**.

Atom–Atom–Atom–Atom	Torsion Angle [°]
O33–S3–C1–Br1	−50.9(2)
O33–S3–C1–S2	−167.8(2)
O33–S3–C1–S1	66.5(2)
O32–S3–C1–Br1	67.4(2)
O32–S3–C1–S2	−49.5(2)
O32–S3–C1–S1	−175.3(2)
O22–S2–C1–Br1	−46.7(2)
O22–S2–C1–S3	69.8(2)
O22–S2–C1–S1	−164.8(2)
O21–S2–C1–Br1	−167.0(2)
O21–S2–C1–S3	−50.5(2)
O21–S2–C1–S1	74.9(2)
O12–S1–C1–Br1	−49.1(2)
O12–S1–C1–S2	69.2(2)
O12–S1–C1–S3	−166.5(2)
O31–S3–C1–Br1	−172.4 (2)
O31–S3–C1–S2	70.7(2)
O31–S3–C1–S1	−55.0(2)
O13–S1–C1–Br1	71.1(2)
O13–S1–C1–S2	−170.6(2)
O13–S1–C1–S3	−46.2(2)
O11–S1–C1–Br1	−167.8(2)
O11–S1–C1–S2	−49.5(2)
O11–S1–C1–S3	74.9(2)
O23–S2–C1–Br1	72.9(2)
O23–S2–C1–S3	−170.5(2)
O23–S2–C1–S1	−45.2(2)

Table S21: Hydrogen bonds in **$K_3[BrC(SO_3)_3] \cdot H_2O$**: Observed donor–acceptor distances (O–O) [pm] and angles (O–H…O) [°].

Donor–Acceptor	Length/	Angle (O-H...O)
O1–O12	287.2(3)	178.1(2)
O1–O13	284.4(4)	154.3(6)
O1–O33	285.6(4)	117.9(5)

Coordination sphere of the cations of the potassium methane(poly)sulfonates

Table S22: Listing of K–O and K-Br distances, coordination numbers and ionic radii observed for the crystallographic unique cations of **$K[Br_3CSO_3] \cdot H_2O$**, **$K_2[Br_2C(SO_3)_2] \cdot H_2O$** and **$K_3[BrC(SO_3)_3] \cdot H_2O$**. The coordination numbers were determined with *Polynator 1.3*.[9]

Compound	Atom	K–O [pm]	K-Br [pm]	Coordination Number	Ionic Radius[7] [pm]
$K[Br_3CSO_3] \cdot H_2O$	**K1**	270.6(5)-317.1(4)	377.5(1)	8	151
	**K2**	271.4(4)-314.5(4)	371.3(1)	8	151
$K_2[Br_2C(SO_3)_2] \cdot H_2O$	**K1**	262.6(2)-294.9(2)	343.1(3)	7	146
	**K2**	266.8(2)-298.6(2)	358.9(6)	8	151
	**K1**	261.1(3)-339.7(3)	369.9(9)	10	159
$K_3[BrC(SO_3)_3] \cdot H_2O$	**K2**	262.9(3)-293.2(3)	352.0(8)-368.9(8)	8	151
	**K3**	260.3(3)-326.6(3)	-	9	155

C Powder XRD

Mo-K$_{\alpha1}$ radiation (λ = 70.930 pm) measurements were prepared in 3.3 borosilicate glass capillaries with an outer diameter of 0.3 mm and glass wall thickness of 0.01 mm. The experimental powder data was measured on a *STOE Stadi P* diffractometer (*STOE & Cie* GmbH, Darmstadt, DE) at room temperature. The reflections were collected on a silicon-based *Dectris Mythen 1K* detector (*Dectris Ltd.*, Baden-Daettwil, CH). The data was processed with *WinX*POW (version 3.12). The Rietveld refinement was done with TOPAS 5.[10]

$K_2[Br_2C(SO_3)_2] \cdot H_2O$

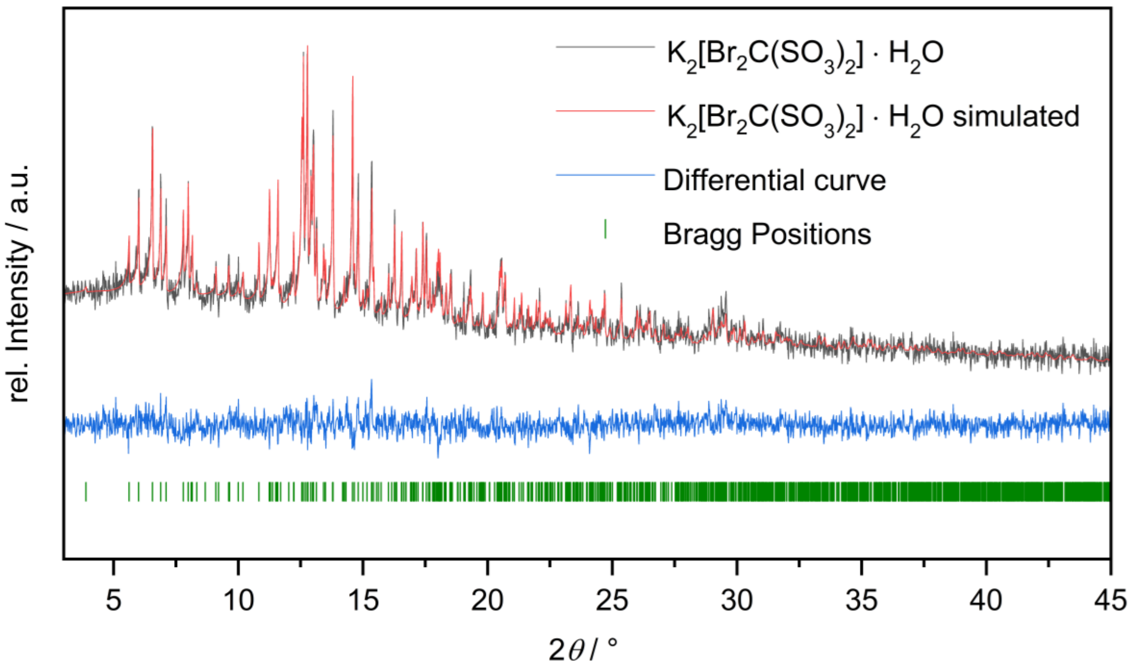

Figure S4: Rietveld refinement plot of **$K_2[Br_2C(SO_3)_2] \cdot H_2O$** (Mo-K$_{\alpha1}$ radiation). Following profile *R*-factors were received: R_{exp} = 3.08%, R_{wp} = 2.79%, R_p = 2.21%, GOF = 0.905. The cell parameter *a*, *b*, *c* and the positions of the two Br atoms were refined. Cell parameters of the refinement are: *a* = 726.37(2) pm, *b* = 715.8(4) pm, *c* = 2094.46(2) pm and *V*= 1.0849(1) nm^3.

$K_3[BrC(SO_3)_3] \cdot H_2O$

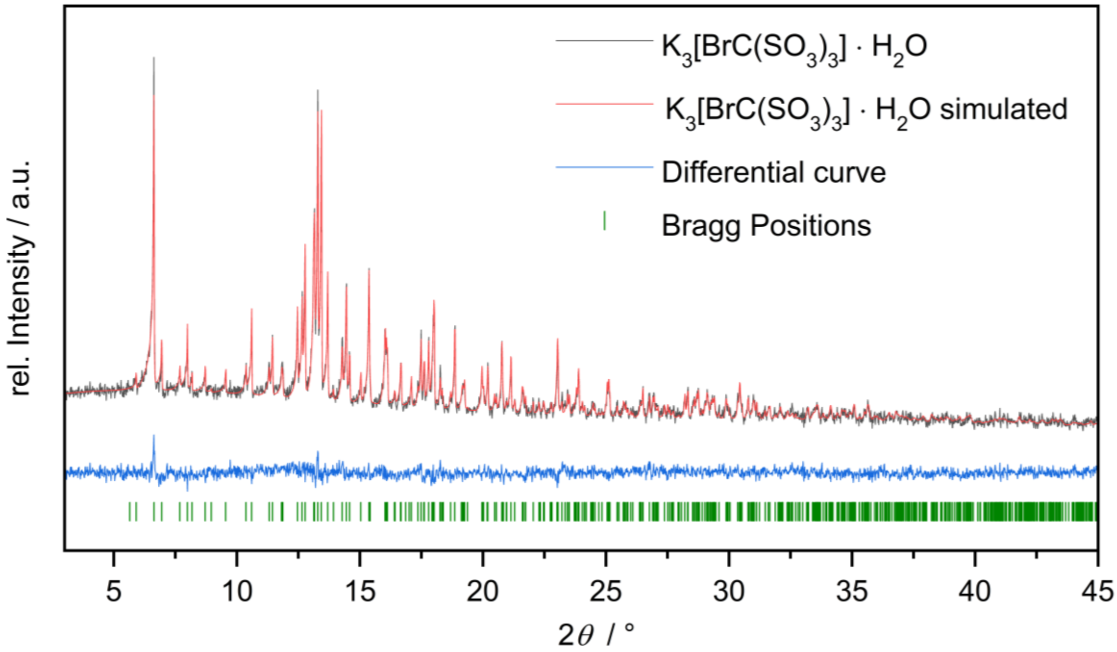

Figure S5: Rietveld refinement plot of **$K_3[BrC(SO_3)_3] \cdot H_2O$** (Mo-$K_{\alpha1}$ radiation). Following profile *R*-factors were received: R_{exp} = 3.88%, R_{wp} = 3.68%, R_p = 2.94%, GOF = 0.948. The cell parameter *a*, *b*, *c* and the positions of the Br atom were refined. Cell parameters of the refinement are: *a* = 719.51(2) pm, *b* = 719.51(2) pm, *c* = 2339.90(1) pm and *V* = 1.2114(1) nm^3.

D Thermal Analysis

The thermal analyses were performed on a thermal analyzer *STA 409C* (*NETZSCH*) coupled with a quadrupole mass spectrometer (*QMS 421*, *Balzers*). All measurements were done in corundum (Al_2O_3) crucibles on a DSC/TG sample holder under an argon flow of 80 ml·min^{-1}. The ion current measurements were carried out in scanning mode with predefined *m*/*z* values. The collected data was processed with *NETZSCH Proteus*.[11]

K[Br$_3$CSO$_3$] · H$_2$O

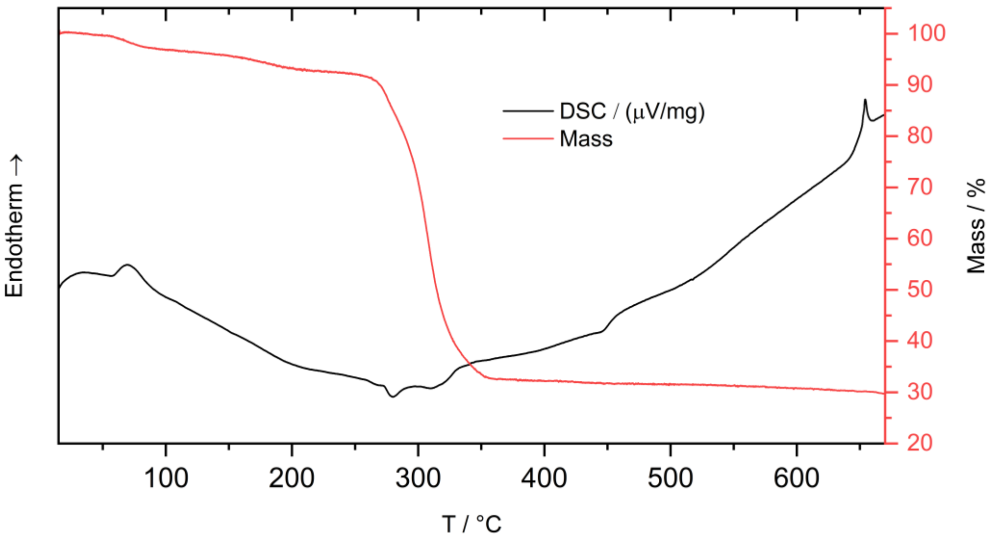

Figure S6: Thermal analysis of **K[Br$_3$CSO$_3$] · H$_2$O** (heating rate 5 K · min^{-1}).

Table S23: Thermal decomposition data of **K[Br$_3$CSO$_3$] · H$_2$O**.

Stage	T_{onset}/ °C	T_{end}/ °C	T_{max}/ °C	Mass loss (calcd.)/ %	Mass loss (obsd.)/ %	Decomposition
**I**	57.02	93.11	69.76	4.49	3.22	Endothermic releasement of H_2O
**II**	249.36	359.98	279.89<br>309.74	62.92	59.92	Decomposition of the potassium tribromomethane-sulfonate to KBr
**Total**				66.78	63.14	Residual phase: KBr

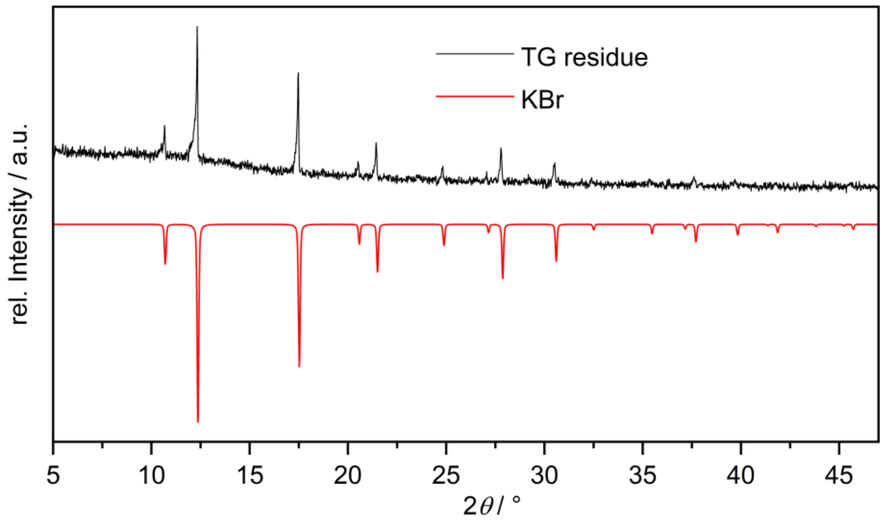

Figure S7: Powder XRD (Mo-K$_{\alpha 1}$) of the residue of the thermal analysis of **K[$Br_3C(SO_3)$] · H_2O** compared to the theoretical pattern of KBr.

$K_2[Br_2C(SO_3)_2] \cdot H_2O$

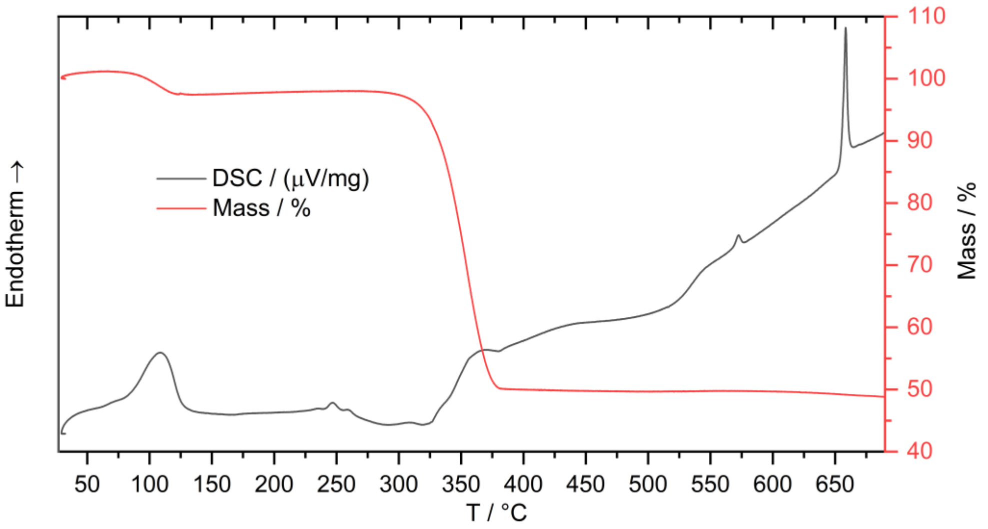

Figure S4: Thermal analysis of $\mathbf{K_2[Br_2C(SO_3)_2] \cdot H_2O}$ (heating rate 5 K · min^{-1}).

Table S24: Thermal decomposition data of $\mathbf{K_2[Br_2C(SO_3)_2] \cdot H_2O}$.

Stage	T_{onset}/ °C	T_{end}/ °C	T_{max}/ °C	Mass loss (calcd.)/ %	Mass loss (obsd.)/ %	Decomposition
**I**	87.25	118.60	108.22	4.15	3.57	Endothermic releasement of H_2O
**II**	299.99	375.55	320.02<br>370.91	47.01	46.26	Decomposition of the potassium dibromomethanedi-sulfonate to K_2SO_4 and KBr
**Total**				51.16	50.01	Residual phase: K_2SO_4, KBr

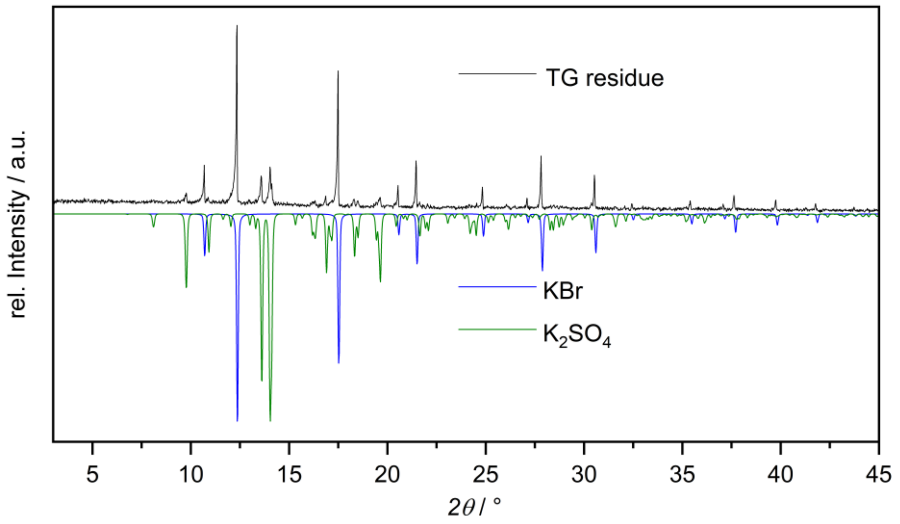

Figure S9: Powder XRD (Mo-K$_{\alpha 1}$ radiation) of the residue of the thermal analysis of $\mathbf{K_2[Br_2C(SO_3)_2] \cdot H_2O}$ compared to the theoretical pattern of α- K_2SO_4 and KBr.

$K_3[BrC(SO_3)_3] \cdot H_2O$

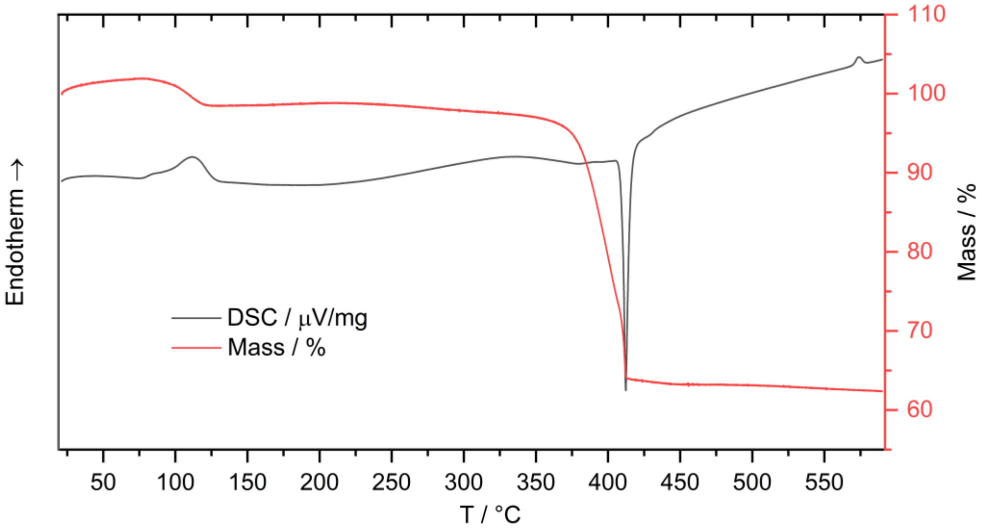

Figure S10: Thermal analysis of **$K_3[BrC(SO_3)_3] \cdot H_2O$** (heating rate 5 K · min^{-1}).

Table S25: Thermal decomposition data of **$K_3[BrC(SO_3)_3] \cdot H_2O$.**

Stage	T_{onset}/ °C	T_{end}/ °C	T_{max}/ °C	Mass loss (calcd.)/ %	Mass loss (obsd.)/ %	Decomposition
I	84.57	120.08	111.45	3.89	3.20	Endothermic releasement of H_2O
II	349.95	436.31	412.29	33.73	33.63	Decomposition of the potassium bromomethanetrisulfonate to K_2SO_4 and KBr
**Total**				37.62	36.83	Residual phase: K_2SO_4, KBr

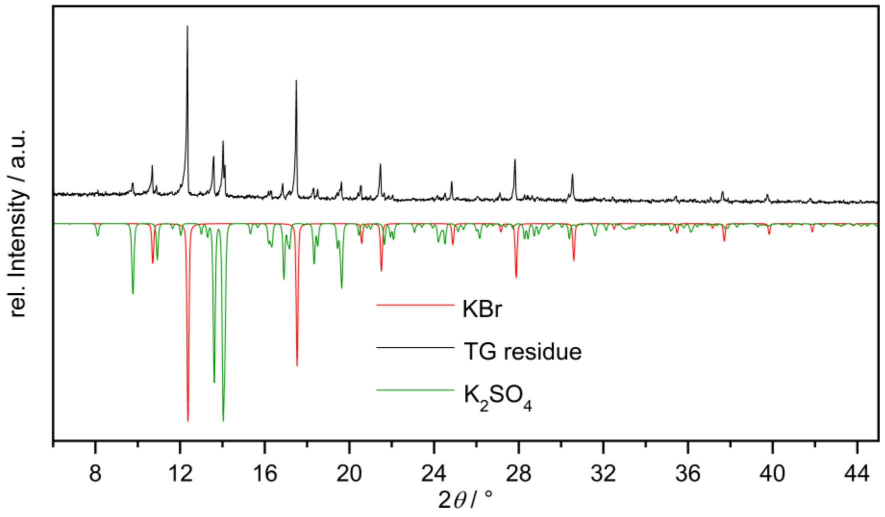

Figure S5: Powder XRD (Mo-K$_{\alpha 1}$ radiation) of the residue of the thermal analysis of **$K_3[BrC(SO_3)_3] \cdot H_2O$** compared to the theoretical pattern of K_2SO_4 and KBr.

E Vibrational Spectroscopy

The Raman spectroscopic data were collected with an *inVia Qotor confocal RAMAN microscope* from *Renishaw GmbH* (Pliezhausen, DE) equipped with 10x, 50x and 100x magnification lenses. The spectra were measured on selected single crystals with a green laser (532 nm, 100 mW) with an exposure time of 10000 ms. The data were processed and corrected by using the WiRE 5.1 software.[12] The simulated spectra were simulated according to the geometry-optimized structures (cf. chapter **F**), according to the procedure described by *Dovesi* et al.[13,14]

$K[Br_3CSO_3] \cdot H_2O$

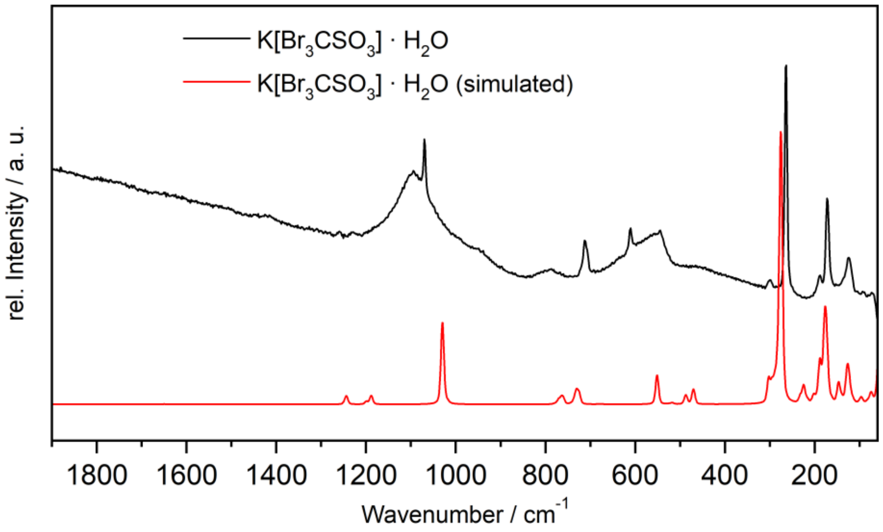

Figure S12: Experimental Raman spectrum of the **$K[Br_3CSO_3] \cdot H_2O$** compared to the theoretical spectrum.

$K_2[Br_2C(SO_3)_2] \cdot H_2O$

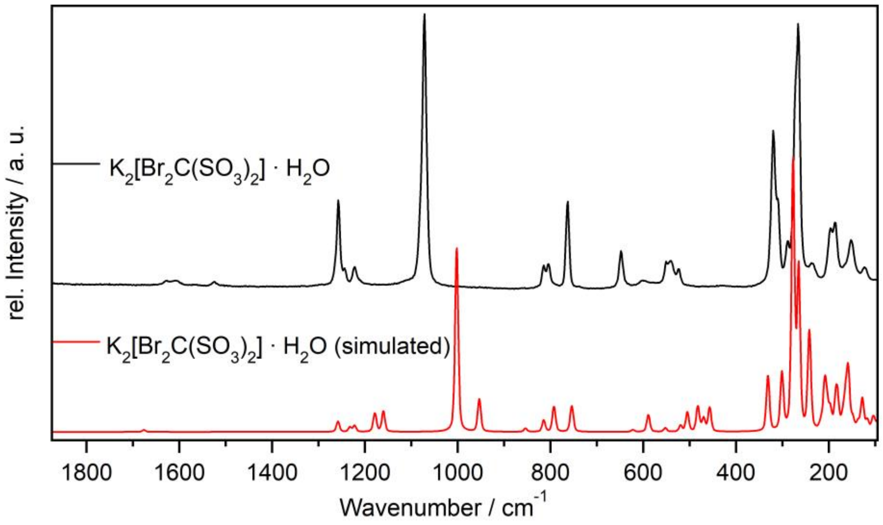

Figure S13: Experimental Raman spectrum of the **$K_2[Br_2C(SO_3)_2] \cdot H_2O$** compared to the theoretical spectrum.

$K_3[BrC(SO_3)_3] \cdot H_2O$

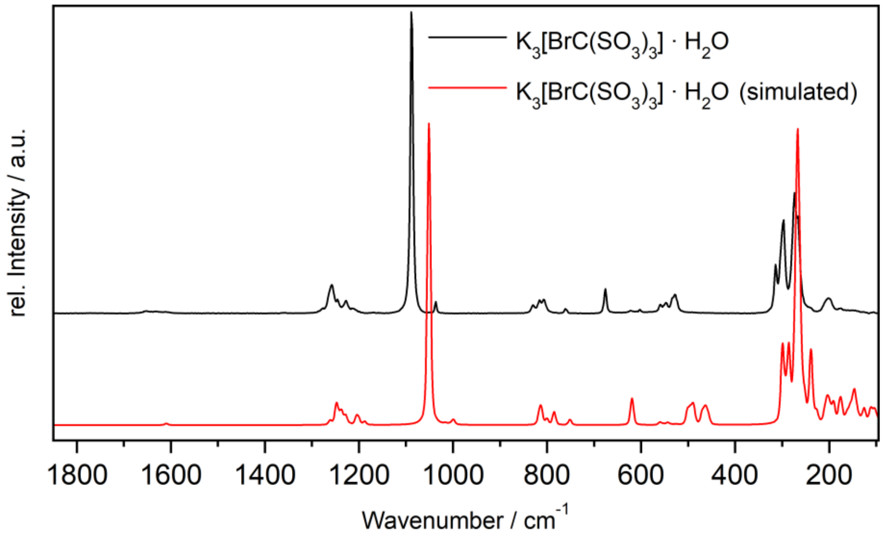

Figure S14: Experimental Raman spectrum of the **$K_3[BrC(SO_3)_3] \cdot H_2O$** compared to the theoretical.

Table S26: Assignment of the Raman bands of the brominated potassium methane(poly)sulfonates.

Assignment	$K[Br_3CSO_3] \cdot H_2O$/ cm^{-1}	Calcd. / cm^{-1}	$K_2[Br_2C(SO_3)_2] \cdot H_2O$/ cm^{-1}	Calcd. / cm^{-1}	$K_3[BrC(SO_3)_3] \cdot H_2O$/ cm^{-1}	Calcd. / cm^{-1}
$\nu_{as}(SO_3)$	1260<br>1231	1244<br>1188	1257<br>1245<br>1222	1258<br>1231<br>1222<br>1178<br>1160	1278<br>1257<br>1246<br>1227	1260<br>1247<br>1237<br>1228<br>1204<br>1188
$\nu_s(SO_3)$	1093<br>1070	1029	1071	1001<br>955	1087<br>1036	1052<br>1000
			814<br>805	814<br>793	830<br>816<br>806	814<br>800<br>784
$\nu(S\text{-}C)$	788	764	763	754	759	752
$\nu_{as}(CBr_3)$	712	729	648	621	675	620
$\delta_s(SO_3)$	610		600	589	622<br>602	561<br>543
$\delta_{as}(SO_3)$	545	551<br>486<br>470	549<br>540<br>524	551<br>520<br>504<br>483<br>470<br>456	559<br>546<br>527	501<br>491<br>463
$\tau(SO_3)$	299	301	320	330<br>301	313<br>297	299<br>286
$\delta_s(CBr_3)$	264	276<br>225	288<br>265<br>237	277<br>264<br>242	273<br>240	267<br>239
$\delta_{as}(CBr_3)$	188<br>172	187<br>175<br>146	196<br>186<br>151	207<br>197<br>183<br>159	201<br>177	203<br>191<br>175<br>146
$\tau(CBr_3)$	123<br>92<br>73	126<br>95<br>75	123	128<br>117		124<br>110

F Results from Quantum Chemical Calculations

K[Br_3CSO_3] · H_2O

Calculation method: A full geometry optimization for K[Br_3CSO_3] · H_2O was performed within density functional theory (DFT) using the PBESOL0 exchange-correlation functional and pob-TZVP-rev2 basis set for all elements.[15-17] The calculations were also used for assigning the Raman frequencies. Throughout the study the *CRYSTAL17* program package was used.[18-21]

Table S27: Crystal data for K[Br_3CSO_3] · H_2O from the geometry optimization.

Empirical formula	$CH_2Br_3KO_4S$
Formula weight	388.899 g · mol^{-1}
Crystal system	triclinic
Space group	$P\bar{1}$ (2)
Unit cell dimensions	a = 678.31 pm
	b = 1047.13 pm
	c = 1287.29 pm
	α = 105.123°
	β = 93.684°
	γ = 90.344°
Volume	0.88061 nm^3
Z	4
Density (calculated)	2.933 g · cm^{-3}

Table S28: Atomic coordinates for K[Br_3CSO_3] · H_2O from the geometry optimization.

**Atom**	***x***	***y***	***z***
Br23	0.8341	0.5244	0.1496
Br21	0.7618	0.2258	0.1503
Br11	0.2961	0.7405	0.1468
Br12	0.3000	1.0481	0.1672
Br22	0.4062	0.3915	0.0867
Br13	-0.0953	0.8770	0.0886
K1	-0.3791	0.8421	0.4203
K2	0.1245	0.6092	0.4195
S1	0.0802	0.9307	0.3214
S2	0.5881	0.4531	0.3179
O22	0.7835	0.4567	0.3811
O11	0.2750	0.9594	0.3869
O13	-0.0271	0.8062	0.3269
O2	0.1533	0.2777	0.4435
O1	-0.3540	1.1872	0.4398
O21	0.4461	0.3452	0.3290
O23	0.4912	0.5849	0.3289
O12	-0.0520	1.0499	0.3362
C1	0.1460	0.8990	0.1780
C2	0.6488	0.3970	0.1732
H1A	0.0861	0.1994	0.3943
H1B	0.2521	0.3069	0.4018
H2A	-0.2525	1.1324	0.4002
H2B	0.4184	1.2331	0.3897

Table S29: Experimental bond lengths [pm] for the **[Br_3CSO_3]$^-$** anion compared with values after geometry optimization by quantum chemical calculations.

**Atom–Atom**	**Experimental Length**	**Calculated Length**
Br23–C2	193.4(5)	192.1
Br21–C2	192.4(5)	191.3
Br11–C1	191.5(5)	191.8
Br12–C1	191.7(5)	191.3
Br22–C2	189.7(5)	191.8
Br13–C1	191.0(5)	191.6
S1–O11	144.3(4)	150.5

S1–O12	145.3(3)	150.9
S1–O13	143.9(3)	152.0
S1–C1	183.5(5)	187.2
S2–O22	144.6(3)	150.5
S2–O21	145.1(4)	152.1
S2–O23	144.4(3)	150.9
S2–C2	184.6(5)	187.5

Symmetry transformations used to generate equivalent atoms: [1]1+X,+Y,+Z; [2]1-X,2-Y,1-Z; [3]-1+X,+Y,+Z; [4]2-X,2-Y,1-Z; [5]2-X,1-Y,1-Z.

Table S30: Experimental bond angles [°] for the **$[Br_3CSO_3]^-$** anion compared to calculated values after geometry optimization by quantum chemical calculations.

Atom–Atom–Atom	Experimental Angle [°]	Calculated Angle [°]
O11–S1–C1	104.3(2)	104.67
O13–S1–O11	114.5(2)	115.22
O13–S1–O12	113.2(2)	113.33
O13–S1–C1	104.6(2)	105.00
O12–S1–C1	104.3(2)	103.48
O22–S2–O21	114.1(2)	113.64
O22–S2–C2	104.5(2)	104.54
O21–S2–C2	104.1(2)	103.08
O23–S2–O22	115.1(2)	115.76
O23–S2–O21	113.1(2)	113.01
O23–S2–C2	104.5(2)	105.13
Br22–C2–Br21	110.6(2)	111.57
S2–C2–Br23	108.0(2)	107.12
Br11–C1–Br12	110.0(2)	111.29
Br13–C1–Br11	110.0(2)	111.12
Br13–C1–Br12	110.4(2)	111.93
S1–C1–Br11	107.9(2)	107.03
S1–C1–Br12	109.5(2)	107.40
S1–C1–Br13	109.1(2)	107.80
Br21–C2–Br23	109.9(2)	111.22
Br22–C2–Br23	110.6(2)	111.59
S2–C2–Br21	109.4(2)	107.98
S2–C2–Br22	108.3(2)	107.12
O23–S2–O22	115.1(2)	115.76

Symmetry transformations used to generate equivalent atoms: [1]1+X,+Y,+Z; [2]-1+X,+Y,+Z; [3]1-X,2-Y,1-Z; [4]2-X,2-Y,1-Z; [5]2-X,1-Y,1-Z.

$K_2[Br_2C(SO_3)_2] \cdot H_2O$

Calculation method: A full geometry optimization for $K_2[Br_2C(SO_3)_2] \cdot H_2O$ was performed within density functional theory (DFT) using the PBESOL0 exchange-correlation functional and pob-TZVP-rev2 basis set for all elements.[15-17] The calculations were also used for assigning the Raman frequencies. Throughout the study the *CRYSTAL17* program package was used.[18-21]

Table S31: Crystal data for $K_2[Br_2C(SO_3)_2] \cdot H_2O$ from the geometry optimization.

Empirical formula	$CH_2Br_2K_2O_7S_2$
Formula weight	428.156 g · mol^{-1}
Crystal system	monoclinic
Space group	*P*2$_1$/*c* (14)
Unit cell dimensions	*a* = 723.93 pm
	b = 713.36 pm
	c = 2112.16 pm
	β = 94.155°
Volume	1.087895 nm^3
Z	4
Density (calculated)	2.614 g · cm^{-3}

Table S32: Atomic coordinates of $K_2[Br_2C(SO_3)_2] \cdot H_2O$ from the geometry optimization.

Atom	*x*	*y*	*z*
Br1	0.5349	0.4582	0.3725
Br2	0.6142	0.8184	0.2895
K1	1.1836	0.6940	0.4904
K2	0.9956	1.1199	0.3246
S2	0.6852	0.8214	0.4342
S1	0.9325	0.5825	0.3523
O23	1.4805	0.8515	0.4406
O1	1.3140	1.1716	0.3957
O22	0.7772	0.7014	0.4870
O11	0.9733	0.4433	0.4057
O13	0.9180	0.4892	0.2880
O12	1.0538	0.7544	0.3580
O21	0.7888	1.0000	0.4226
C1	0.6926	0.6705	0.3621
H1A	1.3858	1.0578	0.4077
H1B	1.2754	1.2220	0.4361

Table S33: Experimental bond lengths [pm] for the $\mathbf{[Br_2C(SO_3)_2]^{2-}}$ anion compared with calculated values after geometry optimization by quantum chemical calculations.

Atom–Atom	Experimental Length	Calculated Length
Br1–C1	193.3(2)	191.9
Br2–C1	193.6(2)	191.2
S2–O23	145(2)	150.2
S2–O22	145.4(2)	151.9
S2–O21	144.8(2)	150.8
S2–C1	184.2(2)	186.9
S1_O11	145.2(2)	151.5
S1–O13	144.9(2)	151.0
S1–O12	144.5(2)	150.8
S1–C1	184(2)	187.2

Symmetry transformations used to generate equivalent atoms: ^{1}1-X,1-Y,1-Z; ^{2}2-X,1/2+Y,1/2-Z; ^{3}-X,1-Y,1-Z; ^{4}-1+X,3/2-Y,1/2+Z; ^{5}-1+X,+Y,+Z; ^{6}1-X,2-Y,1-Z; ^{7}2-X,-1/2+Y,1/2-Z.

Table S34: Experimental bond angles [°] for the the $\mathbf{[Br_2C(SO_3)_2]^{2-}}$ anion compared with calculated values after geometry optimization by quantum chemical calculations.

Atom–Atom–Atom	Experimental Angle [°]	Calculated Angle [°]
O23–S2–O22	113.5(1)	113.73
O23–S2–C1	104.5(1)	104.85
O22–S2–C1	103.66(9	103.72
O21–S2–O23	113.7(1)	114.12
O21–S2–O22	113.4(1)	113.51
O21–S2–C1	106.85(9)	108.03
O11–S1–C1	104.9(1)	105.73
O13–S1–O11	112.7(1)	112.18
O13–S1–C1	104.7(1)	104.00
O12–S1–O11	113.5(1)	113.14
O12–S1–O13	115.0(1)	115.60
O12–S1–C1	104.65(9)	104.95
Br1–C1–Br2	109.5(1)	112.51
S2–C1–Br1	108.4(1)	107.95
S2–C1–Br2	108.7(1)	108.15
S2–C1–S1	112.7(1)	111.34
S1–C1–Br1	108.4(1)	108.20
S1–C1–Br2	109.2(1)	108.72

$K_3[BrC(SO_3)_3] \cdot H_2O$

Calculation method: A full geometry optimization for $K_3[BrC(SO_3)_3] \cdot H_2O$ was performed within density functional theory (DFT) using the PBESOL0 exchange-correlation functional and pob-TZVP-rev2 basis set for all elements.[15-17] The calculations were also used for assigning the Raman frequencies. Throughout the study the *CRYSTAL17* program package was used.[18-21]

Table S35: Structure data for of $K_3[BrC(SO_3)_3] \cdot H_2O$ from the geometry optimization.

Empirical formula	$CH_2BrK_3O_{10}S_3$
Formula weight	467.413 g · mol^{-1}
Crystal system	tetragonal
Space group	$P4_3$ (78)
Unit cell dimensions	a = 716.69 pm
	b = 716.69 pm
	c = 2337.45 pm
Volume	1.200618 nm^3
Z	4
Density (calculated)	2.586 g · cm^{-3}

Table S36: Atomic coordinates of $K_3[BrC(SO_3)_3] \cdot H_2O$ from the geometry optimization.

Atom	*x*	*y*	*z*
Br1	1.2789	1.2250	0.4937
K3	0.8415	0.6080	0.5477
K2	0.5767	1.3909	0.3842
K1	0.9389	0.9426	0.4074
S2	0.6842	1.1345	0.5279
S3	1.4742	0.8796	0.4446
S1	1.3599	0.8868	0.5720
O33	1.2789	0.8304	0.4254
O32	0.5677	1.0086	0.4020
O22	0.7130	1.2869	0.4843
O21	0.8251	0.9789	0.5236
O12	1.3046	1.0329	0.6161
O31	0.5889	0.7115	0.4607
O13	1.1976	0.7765	0.5495
O1	1.0643	0.5252	0.4605
O11	0.5223	0.7686	0.5917
O23	0.6627	1.2113	0.5878
C1	1.4503	1.0270	0.5103

Table S37: Experimental bond lengths [pm] for the $\mathbf{[BrC(SO_3)_3]^{3-}}$ anion compared with calculated values after geometry optimization by quantum chemical calculations.

Atom–Atom	Experimental Length	Calculated Length
Br1–C1	194.7(3)	191.6
S2–O22	144.5(3)	150.8
S2–O21	144.3(3)	150.7
S2–O23	145.0(3)	151.2
S2–C1	185.9(4)	189.0
S3–O33	144.8(3)	151.1
S3–O32	145.6(3)	151.5
S3–O31	144.5(3)	150.6
S3–C1	185.3(3)	187.1
S1–O12	146.3(3)	152.1
S1–O13	144.0(3)	150.1
S1–O11	145.3(3)	151.1
S1–C1	185.7(3)	187.3

Table S38: Experimental bond angles [°] for the **$[BrC(SO_3)_3]^{3-}$** anion compared with calculated values after geometry optimization by quantum chemical calculations.

Atom–Atom–Atom	Experimental Angle [°]	Calculated Angle [°]
O22–S2–C1	106.0(2)	105.63
O22–S2–O23	112.6(2)	112.09
O21–S2–O22	113.2(2)	113.51
O21–S2–O23	113.2(2)	113.52
O21–S2–C1	105.4(2)	106.14
O23–S2–C1	105.6(2)	105.04
O33–S3–O32	111.3(2)	110.90
O33–S3–C1	107.2(2)	106.89
O32–S3–C1	104.7(2)	103.61
O31–S3–O33	113.6(2)	113.14
O31–S3–O32	113.5(2)	114.26
O31–S3–C1	105.8(2)	107.24
O12–S1–C1	104.3(2)	104.03
O13–S1–O12	113.7(2)	113.44
O13–S1–O11	113.9(2)	114.09
O13–S1–C1	105.9(2)	106.30
O11–S1–O12	112.4(2)	112.32
O11–S1–C1	105.4(2)	105.60
S2–C1–Br1	107.7(2)	108.10
S3–C1–Br1	107.3(2)	108.08
S3–C1–S2	110.0(2)	109.11
S3–C1–S1	111.8(2)	111.13
S1–C1–Br1	107.3(2)	109.35
S1–C1–S2	112.5(2)	110.96

G References

[1] K. M. Borys, M. D. Korzyński, Z. Ochal, *Tetrahedron Letters,* **2012**, *53*, 6606–6610.
[2] M. D. Korzyński, K. M. Borys, J. Białek, Z. Ochal, *Tetrahedron Letters,* **2014**, *55,* 745– 748.
[3] F. Van Waes, S. Seghers, W. Dermaut, B. Cappuyns, C. Stevens, *J. Flow. Chem.,* **2014**, *4*, 118–124.
[4] A. Mertens, K. Eppers, D. van Gerven, M. S. Wickleder, *Chem. Eur. J.* **2024**, *30*, e202303617.
[5] Bruker AXS, *Saint V8.40B* **2012**, Madsion, Winconsin, USA.
[6] L. Krause, R. Herbst-Irmer, G. M. Sheldrick, D. Stalke, *J. Appl. Crystallogr.* **2015**, *48*, 3-10.
[7] O. V. Dolomanov, L. J. Bourhis, R. J. Gildea, J. A. K. Howard, H. Puschmann, *J. Appl. Crystallogr.* **2009**, *42*, 339-341.
[8] G. Sheldrick, *Acta Crystallogr. Sec. A* **2015**, *71*, 3-8.
[9] L. Link, R. Niewa, *J. Appl. Cryst.* **2023**, *56*, 1855–1864.
[10] A. Coelho, *J. Appl. Crystallogr.* **2018**, *51*, 210-218.
[11] NETZSCH Gerätebau GmbH, *NETZSCH Proteus 6.1.0* **2019**, Selb, Germany.
[12] Renishaw plc., *WiRE 5.1* **2017**, New Mills, UK.
[13] F. Pascale, C. M. Zicovich-Wilson, F. López Gejo, B. Civalleri, R. Orlando, R. Dovesi, *J. Comput. Chem.* **2004**, *25*, 888-897.
[14] C. M. Zicovich-Wilson, F. Pascale, C. Roetti, V. R. Saunders, R. Orlando, R. Dovesi, *J. Comput. Chem.* **2004**, *25*, 1873-1881.
[15] C. Adamo, V. Barone, *J. Chem. Phys.* **1999**, *110*, 6158-6170.
[16] D. Vilela Oliveira, J. Laun, M. F. Peintinger, T. Bredow, *J. Comput. Chem.* **2019**, *40*, 2364-2376.
[17] J. P. Perdew, A. Ruzsinszky, G. I. Csonka, O. A. Vydrov, G. E. Scuseria, L. A. Constantin, X. Zhou, and K. Burke. Restoring the DensityGradient Expansion for Exchange in Solids and Surfaces. *Phys. Rev. Lett.*, **2008**, *100*, 136406.
[18] R. Dovesi, A. Erba, R. Orlando, C. M. Zicovich-Wilson, B. Civalleri, L. Maschio, M. Rérat, S. Casassa, J. Baima, S. Salustro, B. Kirtman, *WIREs Computational Molecular Science* **2018**, *8*, e1360.
[19] M. Ferrero, M. Rérat, R. Orlando, R. Dovesi, *J. Chem. Phys.* **2008**, *128*, 014110.
[20] M. Ferrero, M. Rérat, R. Orlando, R. Dovesi, *J. Comput. Chem.* **2008**, *29*, 1450-1459.
[21] M. Ferrero, M. Rérat, B. Kirtman, R. Dovesi, *J. Chem. Phys.* **2009**, *129*, 244110.
